# Isolation and characterization of a heavy metal- and antibiotic-tolerant novel bacterial strain from a contaminated culture plate of *Chlamydomonas reinhardtii*, a green micro-alga.

**DOI:** 10.12688/f1000research.53779.2

**Published:** 2021-09-07

**Authors:** Mautusi Mitra, Kevin Manoap-Anh-Khoa Nguyen, Taylor Wayland Box, Taylor Lynne Berry, Megumi Fujita

**Affiliations:** 1 Department of Mathematics, Sciences and Technology, University of West Georgia, Carrollton, Georgia, 30118, USA; 2 Department of Mechanical Engineering, Kennesaw State University, Marietta, Georgia, 30060, USA; 3 Carrollton High School, Carrollton, Georgia, 30117, USA; 4 Department of Chemistry and Biochemistry, University of North Georgia, Dahlonega, Georgia, 30597, USA

**Keywords:** Chlamydomonas, Microbacterium binotii, heavy metal tolerance, Clip185, bioremediation, antibiotic-resistant, Vancomycin-sensitivity, Decaprenoxanthin.

## Abstract

**Background:***Chlamydomonas reinhardtii*, a green micro-alga, is normally cultured in laboratories in Tris-Acetate Phosphate (TAP), a medium which contains acetate as the sole carbon source. Acetate in TAP can lead to occasional bacterial and fungal contamination. We isolated a yellow-pigmented bacterium from a *Chlamydomonas* TAP plate. It was named Clip185 based on the *Chlamydomonas* strain plate it was isolated from. In this article we present our work on the isolation, taxonomic identification and physiological and biochemical characterizations of Clip185.

**Methods:** We measured sensitivities of Clip185 to five antibiotics and performed standard microbiological tests to characterize it. We partially sequenced the 16S rRNA gene of Clip185. We identified the yellow pigment of Clip185 by spectrophotometric analyses. We tested tolerance of Clip185 to six heavy metals by monitoring its growth on Lysogeny Broth (LB) media plates containing 0.5 mM -10 mM concentrations of six different heavy metals.

**Results:** Clip185 is an aerobic, gram-positive rod, oxidase-negative, mesophilic, alpha-hemolytic bacterium. It can ferment glucose, sucrose and mannitol. It is starch hydrolysis-positive. It is very sensitive to vancomycin but resistant to penicillin and other bacterial cell membrane- and protein synthesis-disrupting antibiotics. Clip185 produces a C50 carotenoid, decaprenoxanthin, which is a powerful anti-oxidant with a commercial demand. Decaprenoxanthin production is induced in Clip185 under light. NCBI-BLAST analyses of the partial 16S rRNA gene sequence of Clip185 revealed a 99% sequence identity to that of *Microbacterium binotii* strain PK1-12M and *Microbacterium sp.* strain MDP6. Clip185 is able to tolerate toxic concentrations of six heavy metals.

**Conclusions:** Our results show that Clip185 belongs to the genus *Microbacterium*. In the future, whole genome sequencing of Clip185 will clarify if Clip185 is a new *Microbacterium* species or a novel strain of *Microbacterium binotii*, and will reveal its genes involved in antibiotic-resistance, heavy-metal tolerance and regulation of decaprenoxanthin biosynthesis.

## Introduction

*Chlamydomonas reinhardtii* is a green unicellular alga in the phylum Chlorophyta. It is an excellent experimental system to plant biologists, medical and bioenergy researchers. ^1^^–^^3^ *Chlamydomonas* is normally cultured in the lab in Tris-Acetate-Phosphate (TAP), a medium which contains acetate as the sole carbon source. ^4^ When cultured in TAP, *Chlamydomonas* can perform net biosynthesis of glucose from acetate in TAP via the glyoxylate/C2 cycle, without being strictly dependent on photosynthesis for carbon dioxide fixation. ^5^ Many aerobic bacteria can also utilize the glyoxylate cycle to convert acetate to glucose. ^6^^,^^7^ These aerobic bacteria are capable of growing in TAP. Hence occasionally, we get bacterial contamination on *Chlamydomonas* TAP plates. ^8^^,^^9^

The research presented in this article originated from a high school student research project. This research project was centered on eradicating bacterial contamination from *Chlamydomonas* TAP plates. We observed a yellow-pigmented bacterial contamination on a *Chlamydomonas* strain TAP plate and isolated the bacterium from the contaminated plate. We named this bacterium Clip185 after the *Chlamydomonas* Library Project (CLiP) strain it contaminated. The primary goal of the high school student project was to identify a specific antibiotic and its right concentration that will eliminate Clip185 contamination, without affecting *Chlamydomonas* growth on TAP media plates. This high school microbiology research project was further extended by two undergraduates from the University of West Georgia, to characterize Clip185 on biochemical and physiological levels and determine the genus identity of Clip185.

Clip185 is highly sensitive to vancomycin but is resistant to other microbial cytoplasmic membrane- or translation- disrupting antibiotics. In addition to testing antibiotic-sensitivity of Clip185, we performed several growth analyses and basic microbiological tests to characterize Clip185. It grows better in a Lysogeny Broth (LB) medium than in a TAP medium. Clip185 is an aerobic, mesophilic, alpha-hemolytic, starch hydrolysis-positive, gram-positive bacillus. Clip185 can ferment glucose and sucrose but not lactose. It is a slow fermenter of mannitol compared to *Staphylococcus aureus*, when grown on Mannitol Salt Agar (MSA).

We partially amplified and sequenced the 16S rRNA gene of Clip185. In 2019, NCBI Basic Local Alignment Search Tool ( NCBI-BLAST) analyses of the partial 16S rRNA gene sequence of Clip185 showed 99.10% sequence identity to that of the 16S rRNA gene of *Microbacterium binotii* strain PK1-12M ( Accession #: MN428150.1). In November 2019, we submitted the partial 16S rRNA gene sequence of Clip185 to the NCBI GenBank ( Accession #: MN633284.1). In early 2021, BLAST analyses showed that the best match to the partial 16S rRNA gene sequence of Clip185 is that of *Microbacterium sp.* strain MDP6 ( Accession #: MK128451.1) with 99.33% sequence identity.

The genus *Microbacterium* belongs to the family Microbacteriaceae in the order of Actinomycetales and suborder Micrococcineae. ^10^ *Microbacterium* was first described by Orla-Jensen in 1919. ^11^ In 1983, Collins *et al.* ^12^ rectified the description of the genus *Microbacterium.* In 1998, Takeuchi & Hatano ^13^ fused the closely related genera *Microbacterium* and *Aureobacterium* into the single genus *Microbacterium.* Members of the genus *Microbacterium* have been isolated from diverse environmental sources including soil, water, plants insects, clinical specimens, heavy-metal contaminated sites, deep-sea sediments, dairy products etc. ^10^^,^^14^ Members of the phylum Actinobacteria produce several secondary metabolites like siderophores, antibiotics, and terpenoid pigments that have diverse biological functions, ranging from light absorption, protection against oxidative stress to conferring membrane stability. ^15^

Clip185 is a yellow-pigmented bacterium. Spectrophotometric analyses of the extracted yellow pigment of Clip185 showed that it is a ε-cyclic C _50_ carotenoid, decaprenoxanthin. C _50_ carotenoids are predominantly found in gram-positive Actinomycetales members. ^16^^–^^18^ In many Actinobacteria, decaprenoxanthin biosynthesis is not light-regulated, but in some Actinobacteria decaprenoxanthin biosynthesis is strictly induced under light. ^19^ Light induces decaprenoxanthin biosynthesis in Clip185 like it does in the actinobacterium *Corynebacterium glutamicum*, ^19^ but the induction is weaker than that in *C. glutamicum.*

There are several reports of *Microbacterium sp.* thriving in heavy metal-contaminated environments, ^15^^,^^20^^,^^21^ reducing heavy metals like hexavalent chromium (Cr ^6+^) ^22^^–^^24^ and showing the ability to alter the mobility of heavy metals in contaminated soils. ^25^^,^^26^ We tested Clip185’s ability to tolerate heavy metal stress induced by toxic concentrations of six heavy metals namely zinc (Zn), copper (Cu ^2+^), cadmium (Cd), cobalt (Co ^2+^), nickel (Ni ^2+^) and, hexavalent chromium (Cr ^6+^). 11 of the 16 *Microbacterium* isolates from metal-contaminated soil samples in Indiana, USA, were able to tolerate 0.1 mM of cobalt, cadmium, and nickel and 0.5 mM–20 mM of Cr (VI). ^27^ Four of these 16 *Microbacterium* isolates showed no cadmium tolerence. ^27^ Clip185 can grow in the presence of 6 mM chromium, 2 mM of nickel, cadmium and zinc and 0.5 mM of copper and cobalt in the LB medium. We will have funding in fall 2021 for whole genome sequencing of Clip185 using the PacBio sequel technology. Whole genome sequencing data analyses will not only help us to identify genes in Clip185 that are responsible for antibiotic-resistance, metal tolerance/detoxification and C50 carotenoid production, but will also help to clarify whether Clip185 is a new *Microbacterium* sp. or a new environmental strain of *Microbacterium binotii*. In this article, we present our research on the isolation of Clip185 and its physiological and biochemical characterizations.

## Methods

### Growth media and cultures

*Chlamydomonas* wild type strain 4A+ (CC-4051 4A+ mt+) was cultured in the lab on TAP agar (Chlamydomonas Resource Center) under dim light (15-20 μmol m ^-2^s ^-1^) at 22°C. TAP medium recipes are described in Mitra *et al.*, 2020. ^9^ 4A+ liquid TAP cultures were grown on a shaker under low light (70-80 μmol m ^-2^s ^-1^) for 3 days for aeration as described in Mitra et al, 2020. ^8^^,^^9^ Clip185 stock was maintained in the lab under dim light (15-30 μmol m ^-2^s ^-1^) at 22°C on Lysogeny Broth (LB) agar medium (Cold Spring Harbor Protocols). Experiment-specific temperature and light intensities can be found in the result section of this article under specific experiment descriptions. Clip185 liquid cultures were grown in LB on a shaker at 30°C-37°C for aeration. Light from 1-2 cool white fluorescent lights were used in experiments. A LI-250A light meter (LI-COR, Inc., Lincoln, NE) was used to measure light intensities.

### Testing antibiotic sensitivity of Clip185

We tested the following antibiotics: penicillin; neomycin, chloramphenicol, polymyxin B and vancomycin. Antibiotics were purchased from Sigma-Aldrich (St. Louis, MO). Two different amounts (50 μg and 100 μg) of each of these five antibiotics were tested using the Kirby-Bauer (KB) disc diffusion antibiotic susceptibility tests as described in the American Society for Microbiology (ASM) protocol with one modification: we used TAP agar medium instead of Mueller-Hinton agar medium as stated in the ASM. KB tests of 4A+ and Clip185 were performed on TAP-agar plates as described in Mitra *et al.*, 2020. ^9,^ ^28^^,^^29^ A 12-hours old LB liquid culture of Clip185 was used for plating on the antibiotic-containing TAP plates. Antibiotic plates were incubated at 22°C. Clip185 and *Chlamydomonas* plates were imaged after 7 days of incubation. Diameters of zones of inhibitions were measured in Microsoft PowerPoint by importing free Google ruler available on Chrome. Means and standard deviations were calculated using Microsoft Excel. Microsoft Excels’ t-Test: Paired Two Sample for Means tool in the analysis ToolPak was used for statistical analyses of the data, using three biological replicates per experiment (each with three internal replicates). To test the efficacy of vancomycin as a potent antibiotic in eradicating Clip185 contamination on *Chlamydomonas* TAP plates, Clip185 and 4A+ strains were jointly streaked on TAP media plates containing 50 μg/mL of vancomycin. Streaked TAP + vancomycin plates were imaged after incubation at 22°C for 2.5 weeks. We chose 2.5-weeks incubation period over 7 days in this vancomycin efficacy confirmation experiment to make sure that the selected vancomycin amount is bactericidal to Clip185 and not bacteriostatic.

### Clip185 growth analyses

*Growth at different temperatures*: Clip185 was streaked on LB agar and TAP agar prepared at our laboratory and media plates were incubated at 22°C and 37°C. LB-agar and TAP agar culture plates were imaged after 3 days and 7 days of growth, respectively. Clip185 growth on LB medium plates was monitored for 3 days and that on TAP medium plates was monitored over 7 days because we observed that Clip185 grows slowly in TAP medium compared to that in the LB medium. Three biological replicates per medium per temperature (each with three technical replicates) were used in the experiment.

*Growth assays on Tryptic Soy Blood Agar (TSBA)*: Clip185 and an *Acidovorax sp.*^9^ were streaked on TSBA plates, purchased from Carolina Biological (Burlington, NC). We used a gamma-hemolytic strain of *Acidovorax* as a control in this experiment as it does not hemolyze red blood cells. Plates were incubated at 37°C and were imaged after 3 days of growth. Hemolysis classifications were assigned according to the information stated in the ASM protocol. Three biological replicates per strain (each with three technical replicates) were used in the experiment.

*Growth assays on Mannitol Salt Agar (MSA)*: MSA plates purchased from Carolina Biological (Burlington, NC). Clip185 and *Staphylococcus aureus* (a mannitol fermenter which produces acid, used here as positive control) were streaked on MSA plates, and the plates were incubated at 22°C. The Clip185 MSA plate was imaged after 3-6 days of growth. After 3 days of growth, the *S. aureus* plate was imaged. Three biological replicates per strain (each with three technical replicates) were used in the experiment.

*Growth assays on* T *ris-Phosphate (TP)-phenol red-sugar-agar medium:* TP is the TAP medium without acetate ( https://doi.org/10.17504/protocols.io.bgzujx6w). ^9^ Clip185 was streaked on TP-phenol red-agar medium (pH of 7.2) containing three different sugars as stated under the result section, and the plates were incubated at 22°C. Culture plates were imaged after 7 days of growth. Results were interpreted as described in Mitra *et al.*, 2020. ^9^ Three biological replicates per growth medium (each with three technical replicates) were used in the experiment.

*Comparative growth assays on Potato Dextrose Agar (PDA) and Mueller-Hinton-Agar (MHA)*: Clip185 was streaked on PDA and MHA plates and incubated at 30°C for three days. The plates were imaged after three days of growth. The PDA and MHA plates were purchased from Carolina Biological (Burlington, NC). Three biological replicates per growth medium (each with three technical replicates) were used in the experiment.

*Testing Light-regulation of decaprenoxanthin production:* Clip185, *Corynebacterium glutamicum* strain ATCC13032 and *Kocuria rhizophila* ATCC 9341 strain were streaked on two LB media plates per strain. For each strain, one plate was kept under dim light (20-25 μmol m ^-2^s ^-1^) and the other was kept in the dark at 22°C for five days. Culture plates were imaged after 5 days of growth. Three biological replicates per strain for each light condition (each with three technical replicates) were used in the experiment.

*Growth assays on LB media containing heavy metals*: LB media containing the following concentrations of metal salts were prepared: a) 0.5 mM, 1 mM and 2 mM of cadmium chloride; b) 0.5 mM and 1 mM of cobalt (Co ^2+^) chloride; c) 0.5 mM, 1 mM and 2 mM zinc sulfate heptahydrate; d) 0.5 mM and 1 mM of cupric sulfate pentahydrate; e) 1 mM, 2 mM and 4 mM of nickel (Ni ^2+^) bromide and; f) 2 mM, 4 mM, 6 mM and 10 mM of chromium (Cr ^6+^) standard solution. Cobalt chloride and cadmium chloride salts were purchased from Strem Chemicals (Newburyport, MA). Nickel bromide salt was purchased from Acros organics (Fair Lawn, NJ). Cupric sulfate pentahydrate salt and the chromium (Cr ^6+^) standard solution were purchased from Fisher Scientific (Thermo Fisher Scientific, Waltham, MA). A sterile wooden applicator was lightly tapped over Clip185 growth on a 2-3 days old Clip185 LB-agar plate to collect approximately the same amounts of cells for each experiment. This wooden applicator was used for streaking cells on the respective LB metal plates. The LB metal plates were imaged after a specific period of growth labeled on the figures and also stated in figure legends ( Figures 14- 19). As a control, Clip185 was streaked on a LB plate and the plate was imaged after 8 days and 28 days of growth. All plates were incubated under 15-20 μmol m ^-2^s ^-1^ light intensity. Three biological replicates per metal concentration were used in this experiment. Each biological replicate had three technical replicates.

### Gram staining, oxidase test and starch hydrolysis test

*Gram staining*: Gram staining of Clip185 was performed using commercial Gram stain reagents (VWR, Radnor, PA). Gram-stained Clip185 cells were imaged under an oil immersion lens using a Samsung Galaxy S5 camera and a cell phone adapter for the microscope eyepiece. Three biological replicates (each with three technical replicates) were used in the experiment.

*Cytochrome c oxidase test:* Oxidase test was performed as described in Mitra *et al.*, 2020 ^8, 9^ using Difco DrySlide Oxidase Disposable Slide purchased from Carolina Biological (Burlington, NC). *Sphingobium yanoikuyae* was used as an oxidase-positive control in this experiment. Three biological replicates per strain (each with three technical replicates) were used in the experiment.

*Starch hydrolysis test*: Starch hydrolysis test was performed as described in Mitra *et al.*, 2020 ^8, 9^ using commercial Mueller-Hinton-agar (MHA) medium plates (Carolina Biological, Burlington, NC). Clip185 and *Bacillus subtilis* were streaked on MHA medium plates and incubated for 3 days at 30°C. After 3 days of growth, starch hydrolysis tests were performed. MHA plates were imaged before and after the starch hydrolysis tests. Three biological replicates per strain (each with three technical replicates) were used in the experiment.

### Decaprenoxanthin extractions and spectrophotometric analyses

Actinobacteria like *Corynebacterium glutamicum* ATCC 13032 and *Kocuria rhizophila* ATCC 9341 are known producers of the C _50_ carotenoid decaprenoxanthin. Hence, we selected these two bacterial strains as controls for in the decaprenoxanthin spectrophotometric analyses experiment. Clip185, *C. glutamicum* and *K. rhizophila strains* were grown under a light intensity of 15-20 μmol m ^-2^s ^-1^. Cells of these three strains were treated with 5 mL of 100% methanol for carotenoid extraction. Pigment extraction was carried out in the dark for 6 hours at 22°C. Extracted pigments were processed as described in Mitra *et al.*, 2020. ^8^ Spectrophotometric wavelength (400 nm – 600 nm) scan analyses of extracted pigments were performed using a Beckman Coulter DU 730 Life science UV/Vis spectrophotometer (Brea, CA). Three maximum absorption peaks of decaprenoxanthin were monitored at 413 nm, 437 nm and 467 nm, as described for decaprenoxanthin and its glucosides in the literature. ^30^^–^^32^ *C. glutamicum* and *K. rhizophila* were used as positive controls in spectrophotometric analyses. Three biological replicates per strain were used in this experiment. Each biological replicate had three internal replicates.

### Clip185 genomic DNA isolation and PCR amplification of the partial 16S rRNA gene

Isolation of Clip185 genomic DNA and DNA concentration and purity checking of the isolated genomic DNA were performed as described in Mitra *et al.*, 2020. ^8^^,^^9^ Forward and reverse 16S rRNA PCR primer sequences can be found in Mitra *et al.*, 2020 ^8, 9^ and in Klindworth *et al.*, 2013. ^33^ PCR was performed as described in Mitra *et al.*, 2020 ^8, 9^ with an extension time of 1 minute.

### Gel extraction of the Clip185 16S rRNA amplicon and DNA sequencing

The PCR-amplified partial 16S rRNA Clip185 genomic product (product size approximately 445 bp) was purified from the agarose gel and cloned in pCR4-TOPO TA vector as described in Mitra *et al.*, 2020. ^8^^,^^9^ One clone containing the Clip185 partial 16S rRNA gene was sequenced at the UC Berkeley DNA Sequencing Facility. Analyses of DNA sequences were performed using the Chromas Lite (Technelysium) and BLAST programs.

### Imaging

A Samsung Galaxy S5 cell phone camera was used for imaging all culture plates. Image cropping and adjustments were made using the Photos app in Windows 10 and Adobe Photoshop version 22.3. Visualization and imaging of ethidium bromide -stained DNA gels were performed using a Bio-Rad Molecular Imager Gel Doc XR+ (Bio-Rad, Hercules, CA).

## Results

### Isolation and purification of a yellow-pigmented bacterial strain

**Figure 1.**
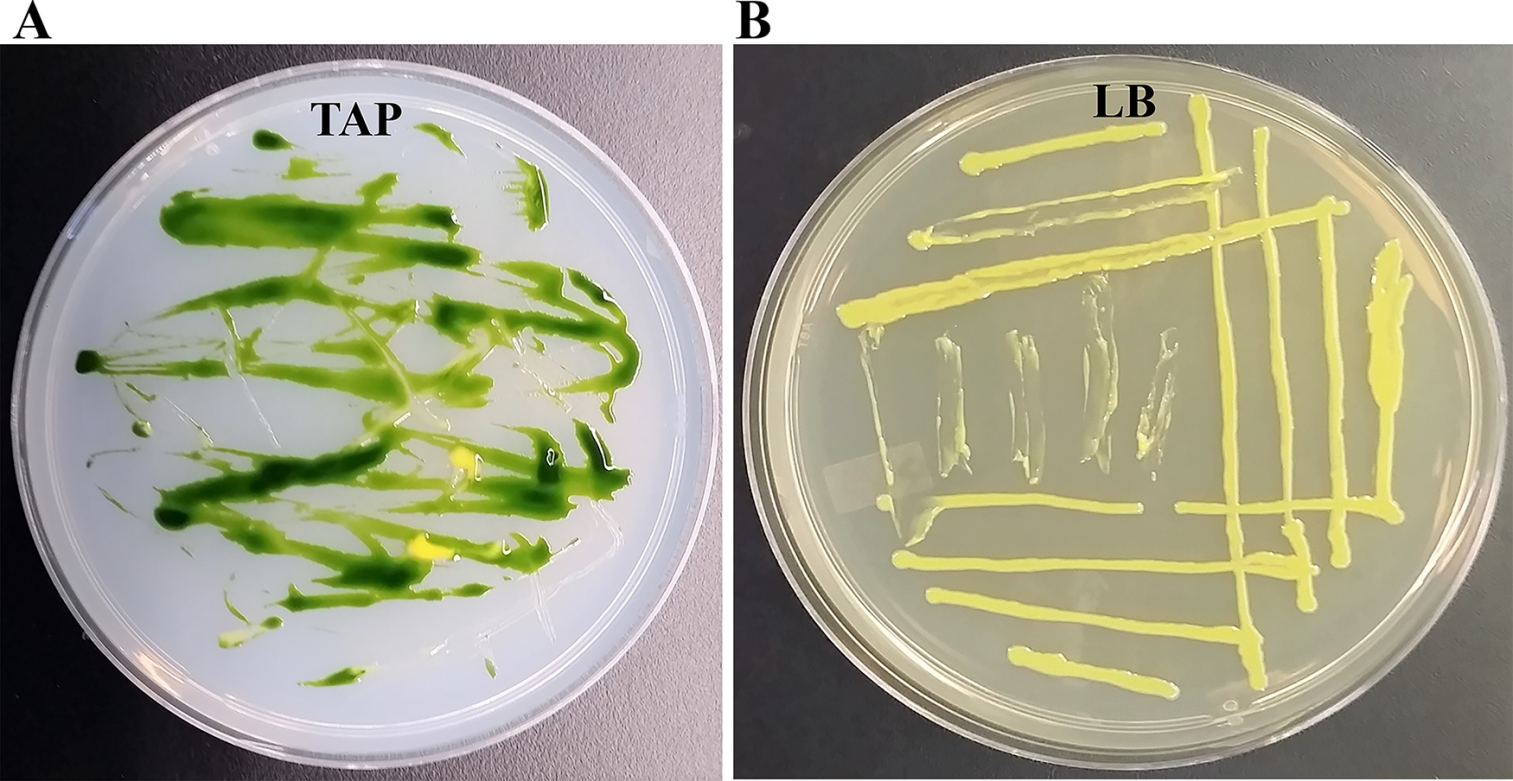
Isolation of the bacterial strain Clip185 from a contaminated *Chlamydomonas* TAP-agar plate. (A) Tris-Acetate-Phosphate (TAP)-agar medium plate showing bacterial contamination of a *Chlamydomonas* strain at room temperature (22°C). (B) LB-agar medium plate of purified Clip185 strain. Culture plate shown in (B) was imaged after 5 days of growth at 22°C under 20-25 μmol m ^-2^s ^-1^ light intensity.

We isolated a yellow-pigmented bacterium from a contaminated TAP media plate of the *Chlamydomonas* strain, CLiP strain LMJ.RY0402. 18514 ( Figure 1A). We purified the bacterium from the contaminated *Chlamydomonas* culture plate using the streak plate method. We picked twenty-one single colonies from Clip185 streaked-LB plate and transferred them to a fresh LB agar plate using a numbered grid (Underlying data ^34^). Colony # 37 was selected for further studies (Underlying data ^34^). We maintained colony # 37 stock on LB agar at 22°C under 15-25 μmol m ^-2^s ^-1^ light intensity ( Figure 1B). We named this bacterium Clip185 after the *Chlamydomonas* Library Project (CLiP) and the first three numerical digits in the second part of the name of the *Chlamydomonas* CLiP strain LMJ.RY0402.185141 it contaminated. We have deposited the Clip185 strain to the ARS Culture Collection (NRRL Accession number: B-65609).

### Clip185 antibiotic susceptibility tests

We monitored relative antibiotic-sensitivities of Clip185 and *Chlamydomonas* 4A+ wild type strain using KB disc diffusion tests as described in Mitra *et al.* 2020. ^8^^,^^9^ In our experiments we used two different amounts (50 μg and 100 μg) of penicillin, chloramphenicol, neomycin, polymyxin B and vancomycin. The mean diameters of zones of growth inhibitions for each antibiotic amount with respective standard deviations are shown in Table 1. For detailed statistical analyses of the data from three biological replicates (each of which had three internal replicates) please refer to our underlying data. ^35^

**Table 1.** Mean diameters of zones of growth inhibitions obtained from disc-diffusion antibiotic susceptibility tests. Zones of growth inhibitions in the presence of five different antibiotics (Penicillin, Chloramphenicol, Neomycin, Polymyxin B and Vancomycin) were studied for *Chlamydomonas reinhardtii* and the bacterial strain, Clip185. Grey and white rows represent 50 μg and 100 μg of each antibiotics applied on the filter paper discs, respectively. Three biological replicates (each had three internal replicates) were used to calculate means and standard deviations shown in the table. Statistical analyses are available as Underlying data. ^35^

Antibiotic	*C. reinhardtii*	Clip185 (Mean ± SD)
Penicillin	0 mm ± 0	0 mm ± 0
	0 mm ± 0	0 mm ± 0
Polymyxin *B*	8.5 mm ± 0.2	0 mm ± 0
	9.6 mm ± 0.4	8.9 mm ± 0.1
Neomycin	9.5 mm ± 0.5	10.6 mm ± 0.1
	11.1 mm ± 0.1	14.8 mm ± 0.2
Chloramphenicol	0 mm ± 0	0 mm ± 0
	0 mm ± 0	0 mm ± 0
Vancomycin	0 mm ± 0	44.5 mm ± 0.15
	0 mm ± 0	52.7 mm ± 0.15

Clip185 and *Chlamydomonas* were both resistant to penicillin and chloramphenicol for both amounts (50 μg and 100 μg) as no zones of growth inhibitions were observed ( Table 1; Underlying data ^35^). *Chlamydomonas* had a larger diameter of zone of growth inhibition for 50 μg polymyxin B (420.15 IU) than Clip185 ( Table 1, Underlying data ^35^). Both *Chlamydomonas* and Clip185 had a larger diameter of zone of growth inhibition for the 100 μg (840.3 IU) polymyxin B compared to that for 50 μg polymyxin B ( Table 1, Underlying data ^35^). P-values from the 1-tailed and 2-tailed hypothesis tests for sensitivity to 100 μg dose of polymyxin B were 3% and 7%, respectively. It is known that if the diameter of the zone of growth inhibition is more than 12 mm with the application of 300 IU of polymyxin B in a KB test, then a bacterium is sensitive to polymyxin B. ^36^ We used polymyxin B amounts that were 1.4-fold to 2.8-fold higher than what is used for KB tests, and growth inhibition zones of *Chlamydomonas* and Clip185 were less than 12 mm ( Table 1). Thus, based on the diameters of the zones of growth inhibitions, both *Chlamydomonas* and Clip185 are resistant to polymyxin B ( Table 1, Underlying data ^35^).

*Chlamydomonas* had a slightly smaller diameter for zone of growth inhibition for 50 μg (38.75 IU) neomycin than Clip185 ( Table 1). P value from the 1-tailed hypothesis test was statistically significant (3%) but the P value from the 2-tailed hypothesis test was not statistically significant for 50 μg neomycin (Underlying data ^35^). Clip185 had a larger dimeter of zone of growth inhibition for 100 μg neomycin (77.5 IU) than *Chlamydomonas* and, the data was statistically significant ( Table 1, Underlying data ^35^). It is known that if the diameter of the growth inhibition zone is more than 16 mm, then a bacterium is sensitive to 30 μg neomycin in a KB test. We used neomycin amounts that were 1.7-fold to 3.3-fold higher than what is used for KB tests ( Table 1), and growth inhibition zones of *Chlamydomonas* and Clip185 were less than 16 mm. Hence both *Chlamydomonas* and Clip185 are resistant to neomycin ( Table 1, Underlying data ^35^). Clip185 was highly sensitive to both 50 μg (50.35 IU) and 100 μg (100.7 IU) vancomycin while *Chlamydomonas* was resistant to vancomycin as it did not show any zones of growth inhibitions ( Table 1, Underlying data ^35^). In summary, our KB test results show that vancomycin is the best drug to minimize Clip185 contamination on TAP agar.

**Figure 2.**
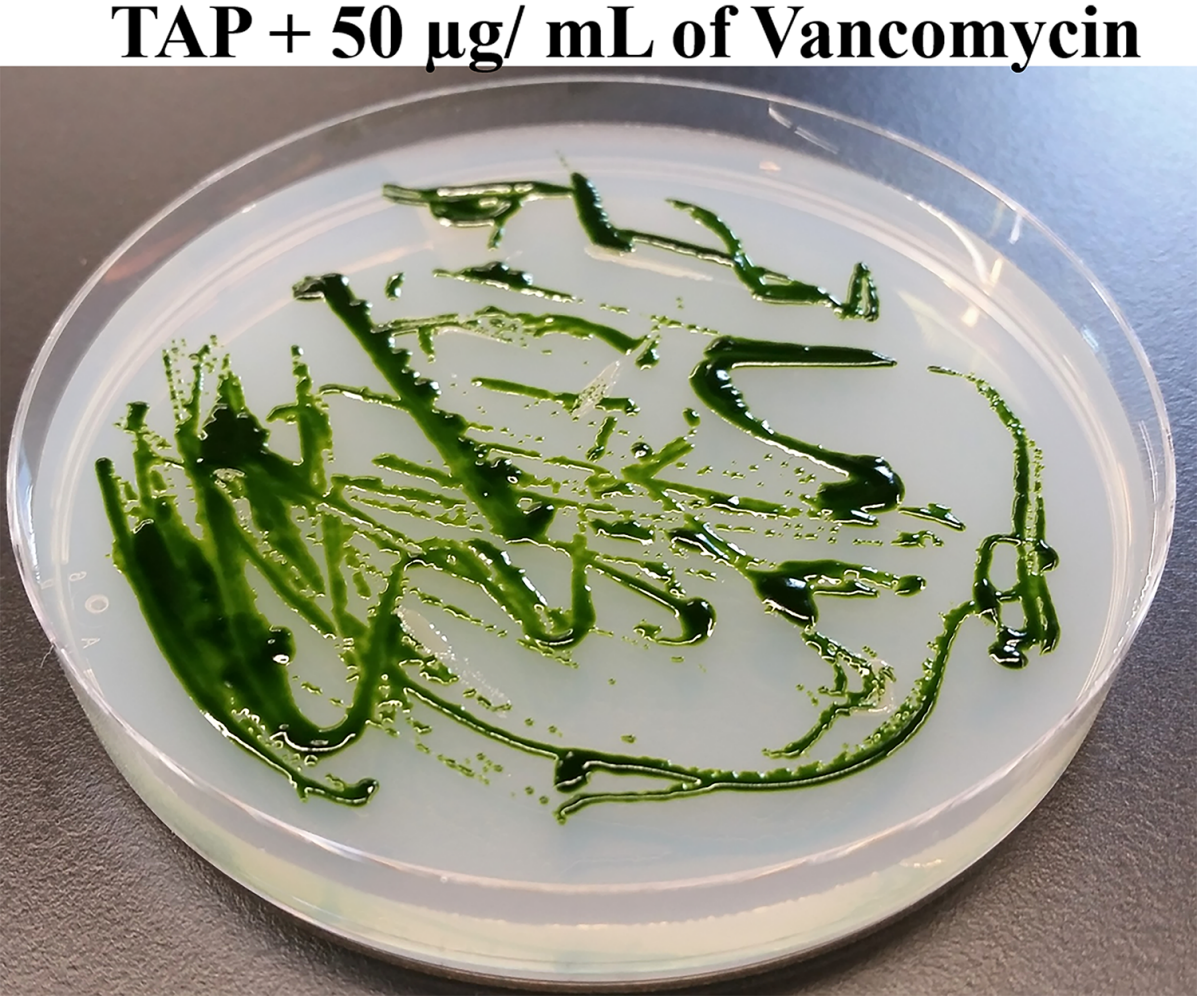
Testing the efficacy of Vancomycin in eliminating Clip185 contamination. *Chlamydomonas* wild type 4A+ and Clip185 strains were streaked on TAP-agar plate containing 50 μg of Vancomycin/mL of medium. TAP-agar antibiotic plate was incubated at room temperature (22°C) for 2.5 weeks before it was imaged.

Next, we streaked *Chlamydomonas* and bacterium Clip185 together on TAP agar plates containing 50 μg of vancomycin per mL of the TAP medium and incubated the plates at room temperature for a period of 2.5 weeks ( Figure 2). Clip185 did not grow on the TAP plate containing 50 μg/mL of vancomycin but *Chlamydomonas* did ( Figure 2) after 2.5 weeks. Hence vancomycin at a concentration of 50 μg/mL in the TAP medium is bactericidal to Clip185 on *Chlamydomonas* culture plates without hindering the algal growth.

### Gram staining

**Figure 3.**
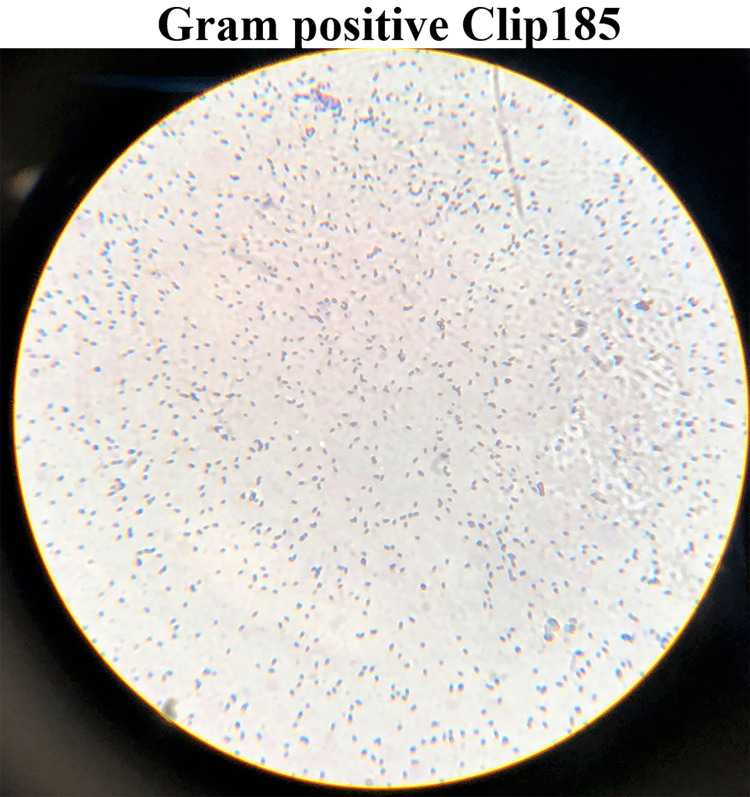
Gram-stained Clip185 under 100× magnification. Clip185 cells from a three days-old LB-agar medium culture plate were used for gram-staining. Gram-stained cells were visualized under an oil immersion lens of a bright-field microscope and imaged with a smart phone camera.

Gram staining revealed that Clip185 is a gram-positive small bacillus. Cells were small rods, and often two rods were joined to each other ( Figure 3).

### Clip185 growth analyses on TAP- and LB-agar medium plates at different temperatures

**Figure 4.**
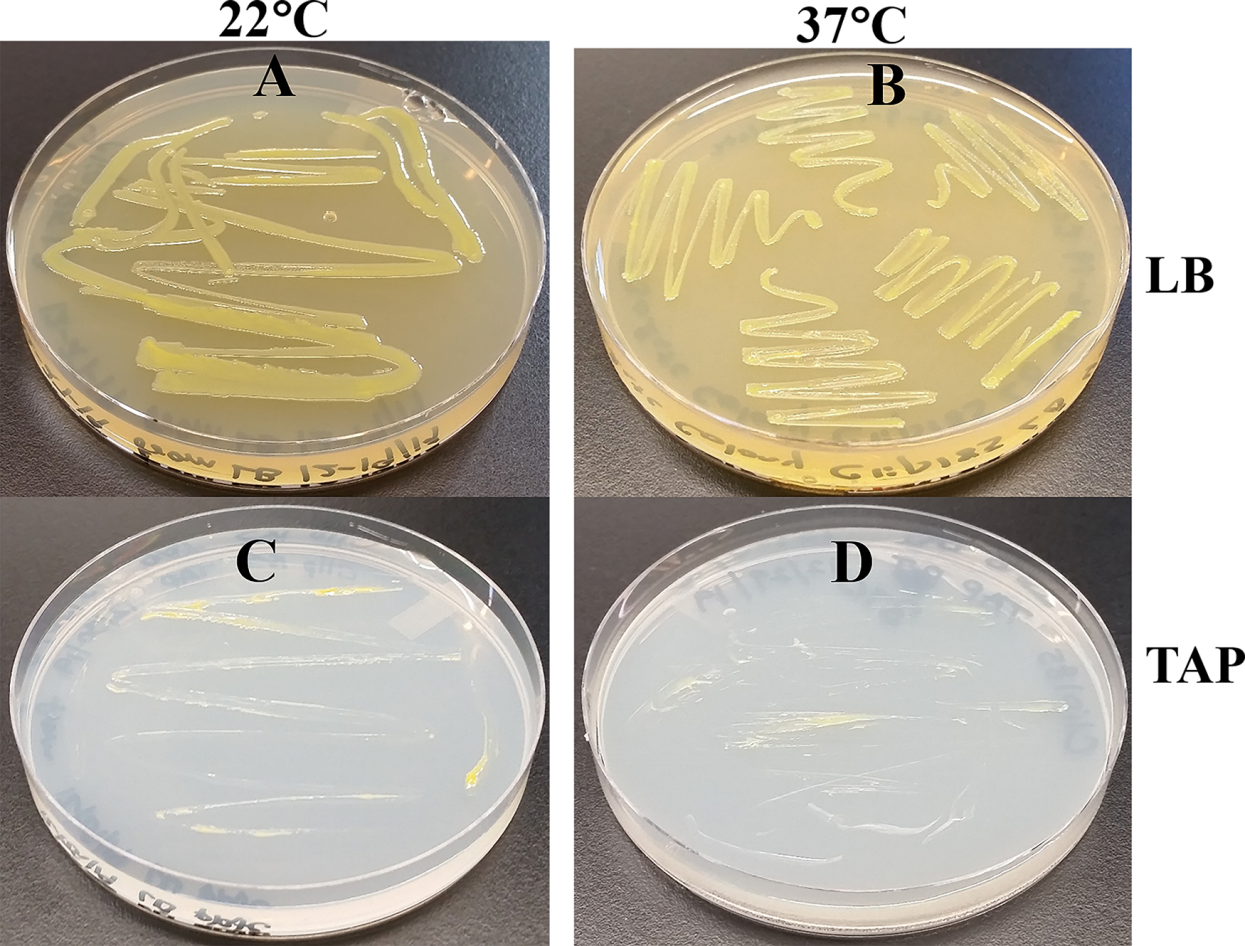
Clip185 growth on TAP- and LB-agar medium at different temperatures. (A) Growth on LB-agar medium plate at room temperature (22°C). (B) Growth on LB-agar medium plate at 37°C. (C) Growth on TAP-agar medium plate at 22°C. (D) Growth on TAP-agar medium plate at 37°C. LB-agar culture plates were imaged after 3 days of growth. TAP-agar culture plates were imaged after 7 days of growth.

Clip185 grew well on LB at 22°C as well at 37°C on LB-agar ( Figure 4A-B). It grew slowly on TAP-agar at 22°C and 37°C ( Figure 4C-D) as evident from the comparison of 3-days growth on the LB plates and 7-days growth on the TAP plates. Hence, Clip185 is a mesophile.

### Testing for Clip185’s ability to lyse red blood cells

**Figure 5.**
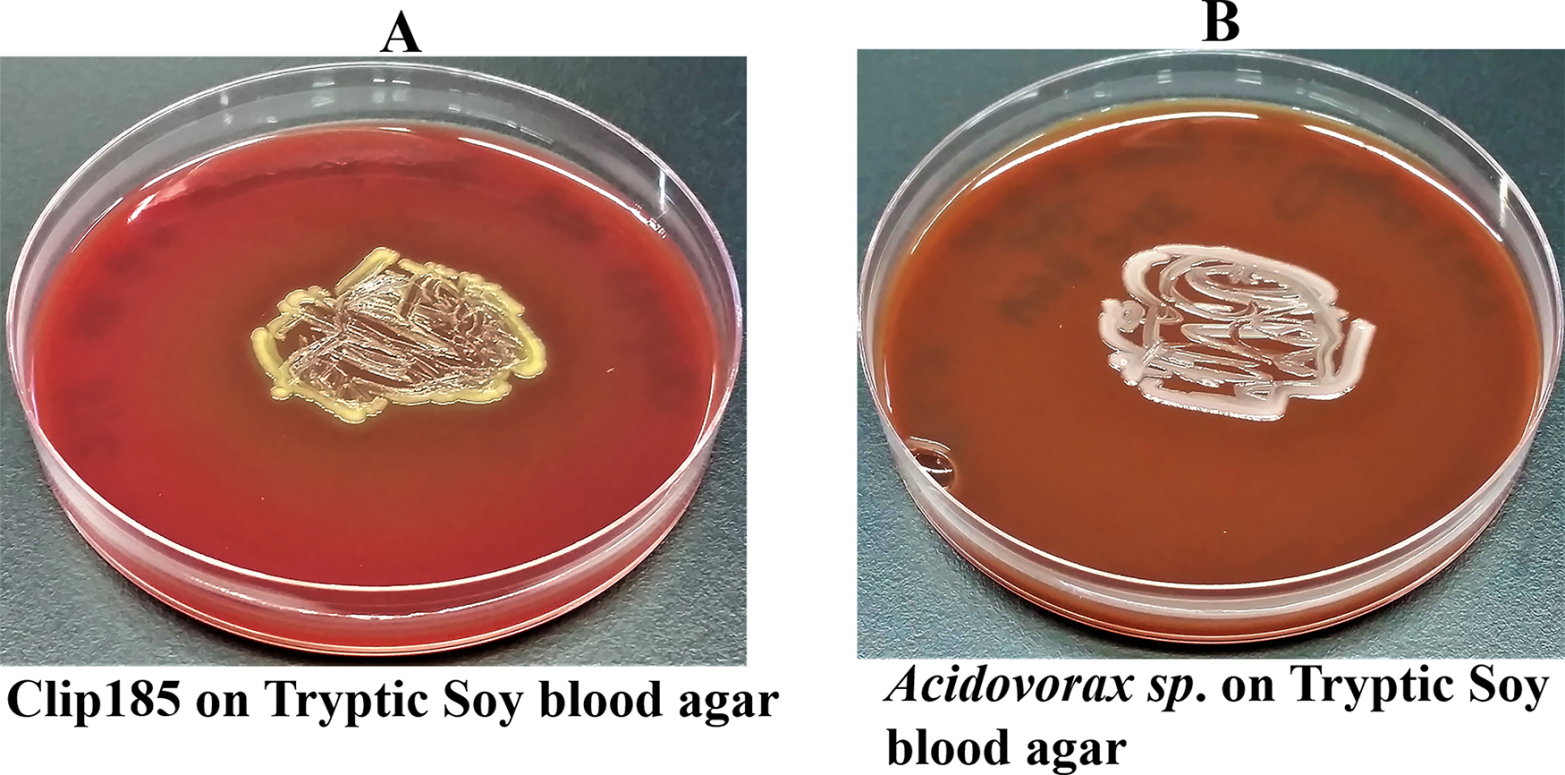
Clip185 is gamma hemolytic. (A) 72 hours-growth of Clip185 on tryptic soy-blood agar plate. (B) 72 hours-growth of a gamma-hemolytic *Acidovorax sp.* on tryptic soy-blood agar plate. Media plates were incubated at 37°C.

Clip185 could partially lyse red blood cells when grown on tryptic soy blood agar plates for three days at 30°C, as evident from the greenish discoloration around the Clip185 growth ( Figure 5A).

We used a gamma-hemolytic strain of *Acidovorax* as a control in this experiment ( Figure 5B) which cannot hemolyze red blood cells. As expected, there was no trace of hemolysis around the growth of *Acidovorax ^9^* on the tryptic soy agar plate ( Figure 5B).

### Testing for presence/absence of cytochrome c oxidase in Clip185

**Figure 6.**
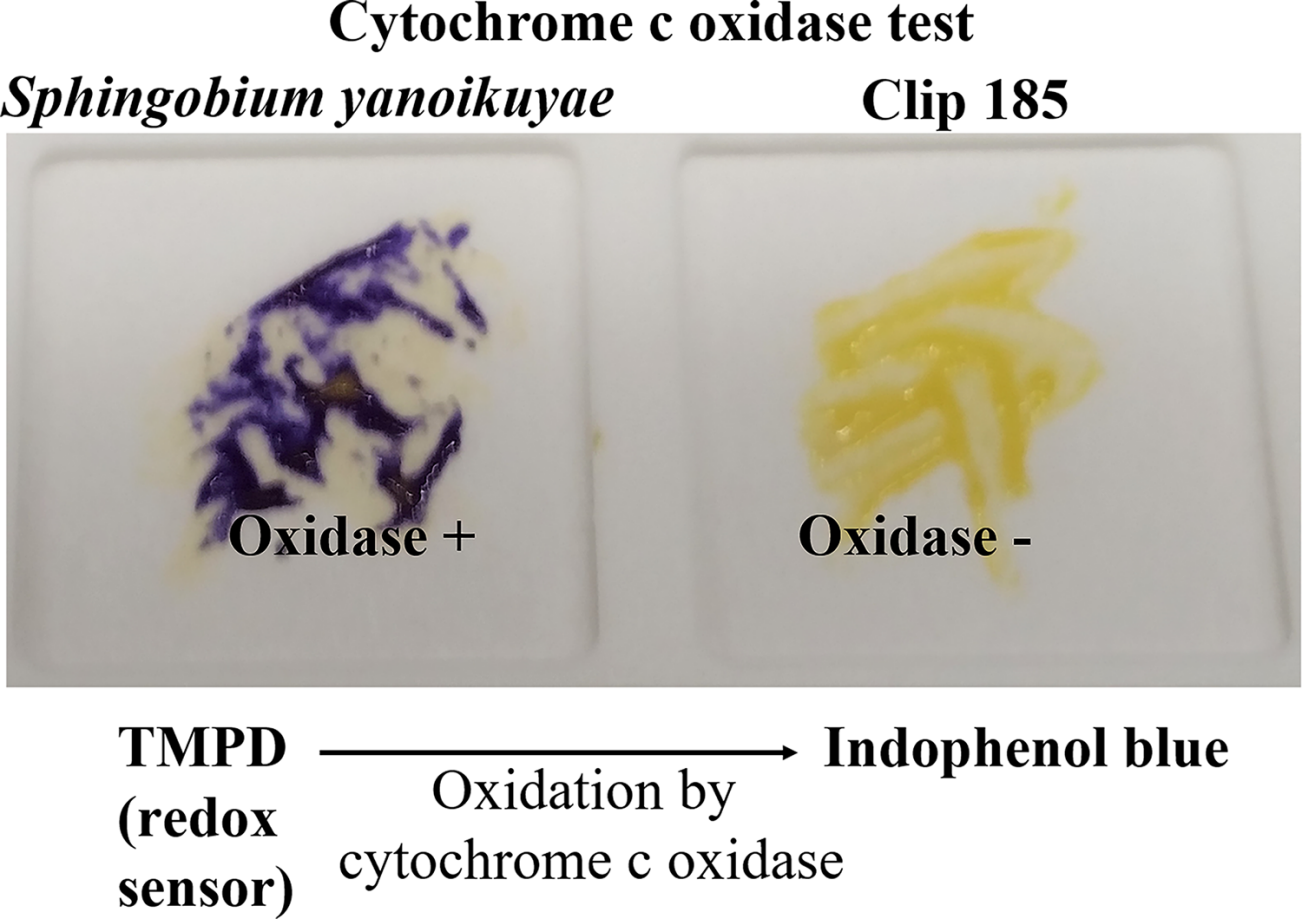
Clip185 is oxidase-negative. Cells of *Sphingobium yanoikuyae* (on the left) and *Clip185* (on the right) streaked on a disposable slide containing a film coated with oxidase reagent tetramethyl-p-phenylenediamine dihydrochloride (TMPD). Image of the slide was taken after 10 seconds of the application of the cells on the slide.

We conducted oxidase test on Clip185 and a gram-negative bacterium *Sphingobium yanoikuyae.* Our oxidase test results showed that *S. yanoikuyae* is oxidase-positive ( Figure 6; left) and Clip185 is oxidase-negative ( Figure 6; right). Results were interpreted as stated under the method section of this article. ^8^^,^^9^ Hence Clip185 does not produce cytochrome c oxidase, an enzyme involved in the electron transport chain in bacterial respiration.

### Testing Clip185’s ability to utilize different sugars as carbon sources and ferment them

**Figure 7.**
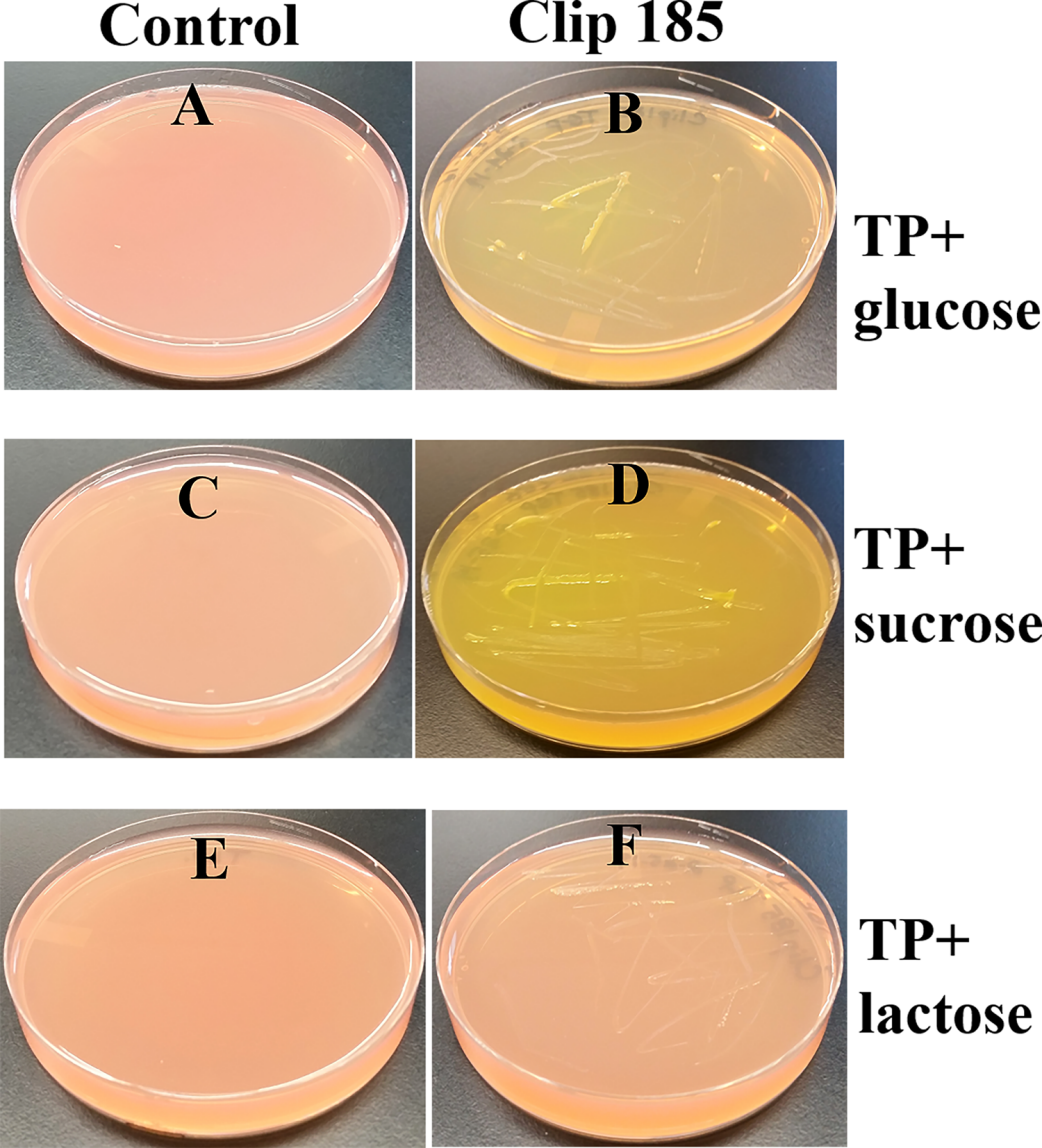
Growth of Clip185 on Tris Phosphate (TP) agar medium supplemented with different sugars. (A) Control TP + 1% glucose agar medium plate with phenol red as a pH indicator. (B) Clip185 on TP +1% glucose agar medium plate with phenol red as a pH indicator. (C) Control TP +1% sucrose agar medium plate with phenol red as a pH indicator. (D) Clip185 growth on TP +1% sucrose agar medium plate with phenol red as a pH indicator. (E) Control TP +1% lactose agar medium plate with phenol red as a pH indicator. (F) Clip185 growth on TP +1% lactose agar medium plate with phenol red as a pH indicator. Culture plates were imaged after 7 days of growth at room temperature.

We did not want to use LB medium to test the ability of Clip185 to utilize other sugars as carbon source, because excess amino acids present in the nutrient-rich LB medium are converted to glucose via gluconeogenesis. TP agar medium is the TAP medium minus acetate. Hence TP medium lacks a carbon source. ^8^^,^^9^ We supplemented TP medium (pH 7.2) separately with three different sugars (glucose, sucrose and lactose) and the pH indicator, phenol red. ( Figure 7). We grew Clip185 on these neutral TP + sugar plates. Figure 7A, C and E represent control TP + 1% glucose, TP + 1% sucrose and TP + 1% lactose plates (without Clip185), respectively. These control plates show the light reddish color of phenol red when the pH is 7.2. Clip185 grew on TP + 1% glucose and TP +1% sucrose plates and fermented the respective sugars ( Figure 7B; Figure 7D) but it did not grow on TP + 1% lactose plate ( Figure 7F). Fermentation of glucose and sucrose produced acids which decreased the pH in the TP medium. With the decrease in pH, phenol red’s light red color changed to a yellow color ( Figure 7B; Figure 7D).

### Clip185’s ability to grow on Mannitol Salt Agar (MSA) and ferment mannitol

**Figure 8.**
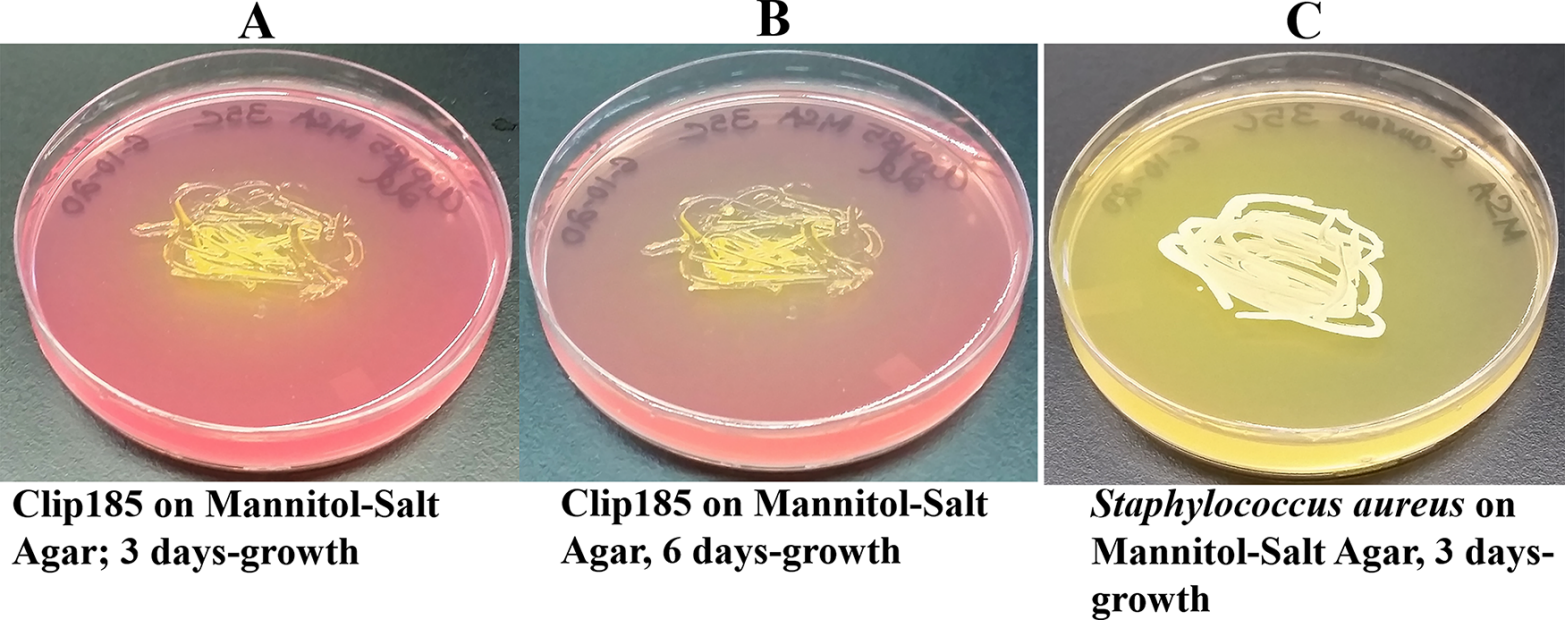
Clip185 growth on Mannitol Salt Agar (MSA) medium. (A) 3 days-growth of Clip185 on MSA plate. (B) 6 days-growth of Clip185 on MSA plate *.* (C) 3 days-growth of *Staphylococcus aureus* on MSA plate. Media plates were incubated at 30°C.

Clip185 grew on MSA ( Figure 8A-B) and fermented mannitol in MSA to acid slowly compared to the fast mannitol fermentation displayed by the gram-positive bacterium. *Staphylococcus aureus* ( Figure 8C). Acid production decreased the pH of MSA. With the decrease in pH, phenol red’s light red color changed to a yellow color ( Figure 8).

### Starch hydrolysis test

Starch has two forms: linear amylose and branched amylopectin. The large sizes of amylose and amylopectin prevent these chemicals from being transported across the bacterial cell wall. Bacteria secrete out the α-amylase and oligo-1,6-glucosidase enzymes to degrade starch. These enzymes degrade extracellular starch into glucose/dextran which can be utilized by bacteria as carbon sources. A starch hydrolysis test is used to identify bacteria that can utilize extracellular starch as a carbon/energy source. Traditionally, potato dextrose agar (PDA) is used for starch hydrolysis tests. Clip185 did not grow well on PDA (Underlying data ^37^). It grew well on Mueller-Hinton Agar (MHA), which is a rich medium used for growing bacteria that have special nutritional requirements (fastidious bacteria) (Underlying data ^37^). Hence, we chose MHA, which contains 0.15 % starch, to test Clip185’s ability to hydrolyze starch.

**Figure 9.**
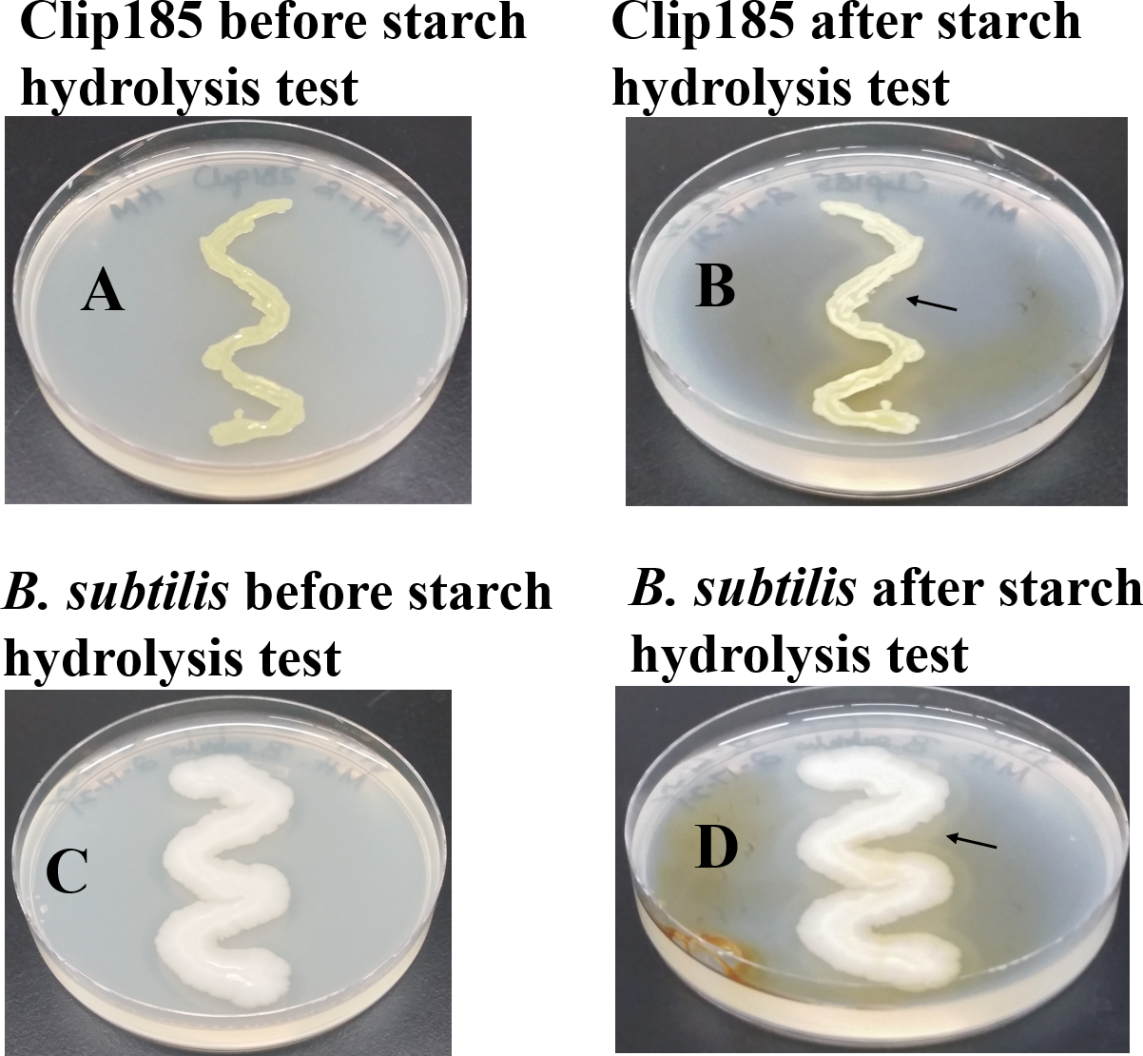
Clip185 can hydrolyze starch. (A) 3 days -growth of Clip185 at 30°C on Mueller-Hinton medium which contains 0.15% starch. (B) Clip185 Mueller-Hinton plate shown in (A) was treated with Gram iodine. (C) 3 days-growth of *Bacillus subtilis* at 30°C on Mueller-Hinton medium which contains 0.15% starch. (D) *B. subtilis* Mueller-Hinton plate shown in (C) treated with Gram iodine. Black arrows indicate the clear starch-hydrolyzed zones around the bacterial growth.

We used *Bacillus subtilis,* which is known to hydrolyze starch, as a positive control in the experiment. We grew both Clip185 and *B. subtilis* on MHA for 3 days and imaged the plates ( Figure 9A; Figure 9C). Gram iodine was then added to the both Clip185 and *B. subtilis* plates. Iodine reacted with the starch in MHA to produce a light blue color. Both Clip185 ( Figure 9B) and *B. subtilis* ( Figure 9D) tested positive in the starch hydrolysis test as there is a visible clear zone indicated by the black arrows, surrounding the growth of each of these two strains on the MHA due to the hydrolysis of starch.

### Partial 16S rRNA gene sequencing of Clip185

**Figure 10.**
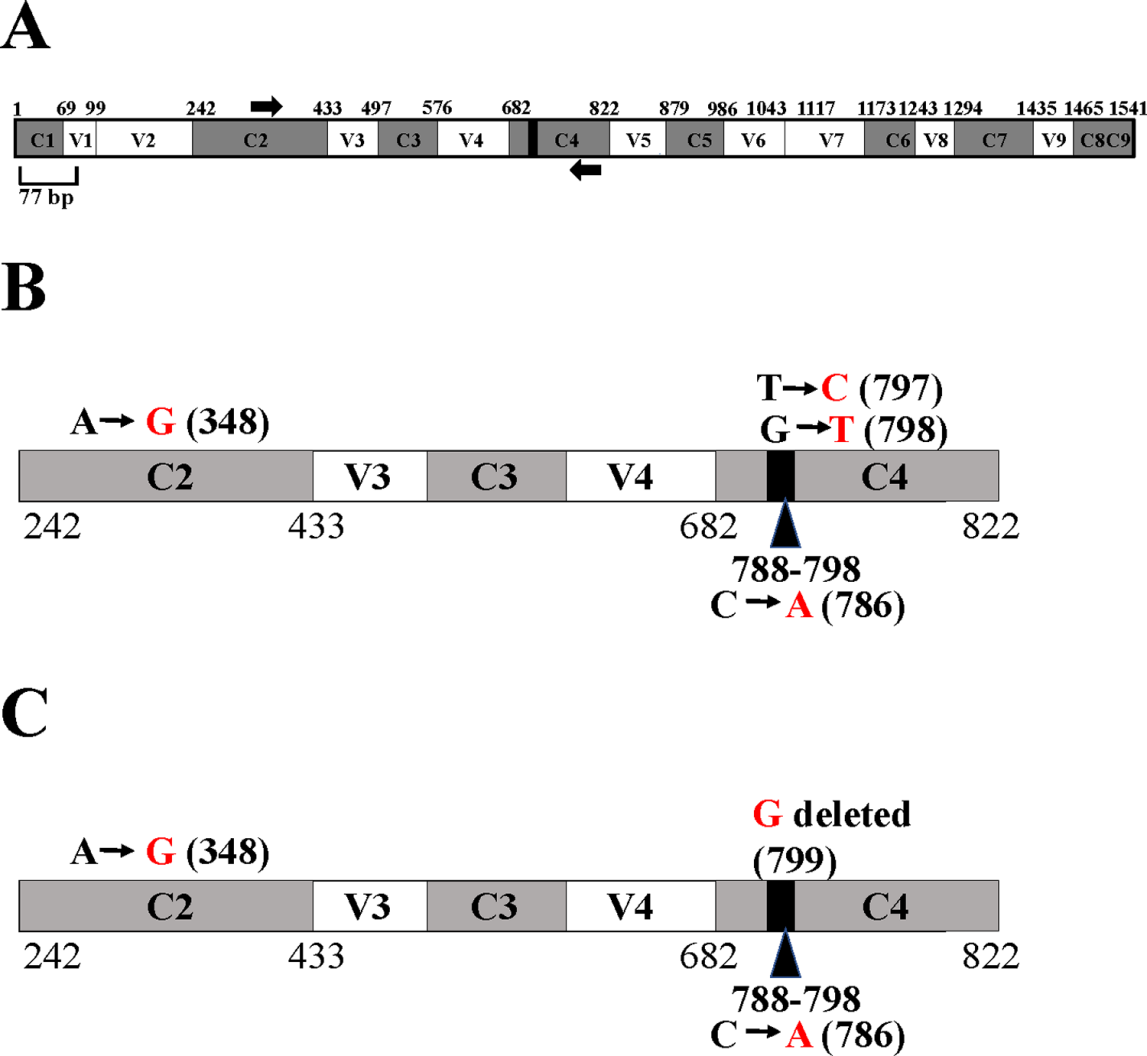
PCR amplification of the partial 16S rRNA gene of Clip185 and NCBI-BLAST analyses. (A) A schematic diagram showing the nine conserved and hypervariable regions in the 16S rRNA gene. The interspersed conserved regions (C1– C9) are shown in dark gray, and the hypervariable regions (V1–V9) are depicted in white. The black box within the C4 region represents 11 nucleotides (788-798 base pairs) that are invariant in bacteria. ^8^^,^^9^ Thick black arrows denote 16S rRNA gene-specific forward and reverse PCR primers in the C2 region and in the C4 region, respectively. The gene diagram is based on the 16S rRNA gene sequence of *E. coli.* See also Underlying data. ^42^ (B) A schematic diagram showing the nucleotide changes in Clip185 in the 16S rRNA region spanning the C2 and C4 regions in comparison to the best NCBI- BLAST hit in 2019 (score of 800; E-value 0 and percent identity of 99.10%): *Microbacterium binotii* strain PK1-12M 16S ribosomal RNA gene, partial sequence (GenBank Accession #: MN428150.1). (C) A schematic diagram showing the nucleotide changes in Clip185 in the 16S rRNA region spanning the C2 and C4 regions in comparison to the best NCBI- BLAST hit in 2021 (score of 806; E-value 0 and percent identity of 99.33%): *Microbacterium sp.* strain MDP6 16S ribosomal RNA gene, partial sequence (GenBank Accession #: MK128451.1). Black nucleotides show the native nucleotides in the BLAST hits *Microbacterium binotii* strain PK1-12M and *Microbacterium sp.* strain MDP6 that were substituted by the depicted red nucleotides in Clip185 16S rRNA gene sequence. The black bold numbers within the parenthesis beside the nucleotides show the specific nucleotide positions where the nucleotide changes have occurred. Nucleotide positions shown in the figures have been assigned according to that of the 16S rRNA gene sequence of *E. coli.*

The 16S rRNA gene has nine hypervariable (V1-V9) and nine conserved regions (C1-C9). ^38^^–^^40^ We used the *Escherichia coli* 16S rRNA gene which is 1541 bp long ( Figure 10A), as a reference in the 16S rRNA gene schematics in Figure 10. A total of 11 nucleotides (788-798) located within the C4 conserved region are completely conserved in bacteria. ^41^ The 11 bp super-conserved region is represented by the black box within the C4 region in the schematics ( Figure 10). The forward and reverse 16S rRNA gene-specific PCR primers are represented by black arrows in the 16S rRNA gene schematics ( Figure 10A). A 445 bp amplicon was generated upon PCR amplification of the partial 16S rRNA gene of Clip185 (Underlying data ^42^). This 445 bp amplicon was cloned. A clone harboring the Clip185 amplicon was then sequenced to determine the taxonomic identity of Clip185.

In 2019, NCBI-nucleotide BLAST analyses detected *Microbacterium binotii* strain PK1-12M ( GenBank Accession #: MN428150.1) as the nearest match to Clip185 based on the partial 16S ribosomal RNA gene sequence, with a score of 800, an E-value of 0 and percent identity of 99.10%. Four nucleotide substitutions were identified in Clip185’s 16S rRNA partial gene sequence relative to that in *Microbacterium binotii* strain PK1-12M ( Figure 10B). In the C4 region, one transition and one transversion were detected ( Figure 10B). We found one transition in the C2 region and one transversion in the 11 bp super-conserved sub-region within C4 region ( Figure 10B). We deposited this partial 16S rRNA gene sequence of Clip185 in NCBI GenBank with the description: *Microbacterium binotii* strain PK1-12M variant 16S ribosomal RNA gene, partial sequence ( Accession number: MN633284.1). In early 2021, BLAST analyses detected a new match: *Microbacterium sp.* strain MDP6 ( Accession #: MK128451.1) with a score of 806, E-value of 0 and percent identity of 99.33%) (NCBI last accessed 8-26-21, Figure 10C). We detected three nucleotide changes in the 16S rRNA gene of Clip185 with respect to this new hit: one transition in the C2 region, one transversion in the 11 bp super-conserved sub-region within C4 region and one deletion in the C4 region ( Figure 10C). Analyses of available genome sequences of *Microbacterium sp.* on NCBI revealed that whole genome sequences for *Microbacterium binotii* strain PK1-12M and *Microbacterium sp.* strain MDP6 are not available (NCBI data last accessed on 8-26-2021).

### Spectrophotometric analyses of pigments synthesized by Clip185

**Figure 11.**
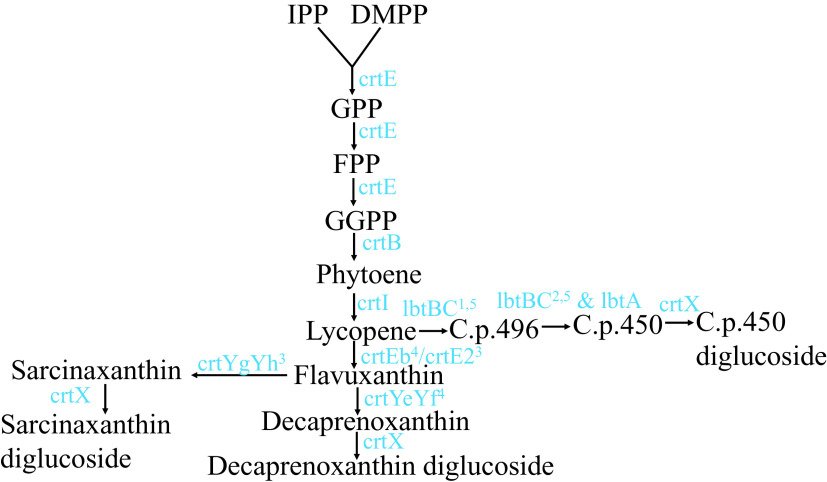
A schematic representation of the C _**50**_ biosynthesis pathways in bacteria. C _40_ carotenoid lycopene is diprenylated to form an acyclic C _50_ carotenoid, flavuxanthin. Flavuxanthin undergoes cyclization in three different ways to form three types of natural C _50_ carotenoids in bacteria. Substrates and intermediate products names are abbreviated and are shown in black. Genes encoding enzymes catalyzing specific reactions/steps in the pathways are shown in blue. Abbreviations: IPP, isopentenyl pyrophosphate; DMPP, dimethylallyl pyrophosphate; GPP, geranyl pyrophosphate; FPP, farensyl pyrophosphate; GGPP, geranylgeranyl pyrophosphate; C.p. 496, 2,2′-Bis-(3-methylbut-2-enyl)-3,4,3′,4′-tetradehydro-1,2,1′,2′-tetrahydro-ψ, ψ-carotene-1, 1-diol; C.p.450, 2,2′-Bis-(4-hydroxy-3- methybut-2-enyl)-β, β-carotene. Superscripted numbers: 1 denotes lbtC in the lbtBC fused gene; 2 denotes lbtA and lbtB in the lbtBC fused gene; 3 denotes *Micrococcus luteus* gene *;* 4 denotes *Corynebacterium glutamicum* genes and 5 denotes *Dietzia sp.* CQ4 genes *.*

Carotenoids are synthesized from the precursor isopentenyl pyrophosphate and its isomer dimethylallyl pyrophosphate in the terpenoid biosynthetic pathway ^16^ ( Figure 11). Two molecules of geranylgeranyl diphosphate condense to form phytoene ( Figure 11). Lycopene (a red C _40_ carotenoid) is formed by the four-step enzymatic desaturation of phytoene ( Figure 11). Lycopene is diprenylated and hydroxylated to form C _50_ carotenoids ^16^^,^^17^ ( Figure 11). To date, three different C _50_ carotenoid biosynthetic pathways are known in the literature: 1) the ε-cyclic C _50_ carotenoid decaprenoxanthin biosynthetic pathway in *Corynebacterium glutamicum*; ^16, 43, 44^ 2) the β-cyclic C _50_ carotenoid C.p.450 biosynthetic pathway in *Dietzia sp.* CQ4 ^45^ and; 3) the γ-cyclic C50 carotenoid sarcinaxanthin biosynthetic pathway in *Micrococcus luteus* NCTC2665. ^18^ *Microbacterium* belongs to the gram-positive order of Actinomycetales. Many members of Actinomycetales produce the C _50_ carotenoid, decaprenoxanthin. ^16^

**Figure 12.**
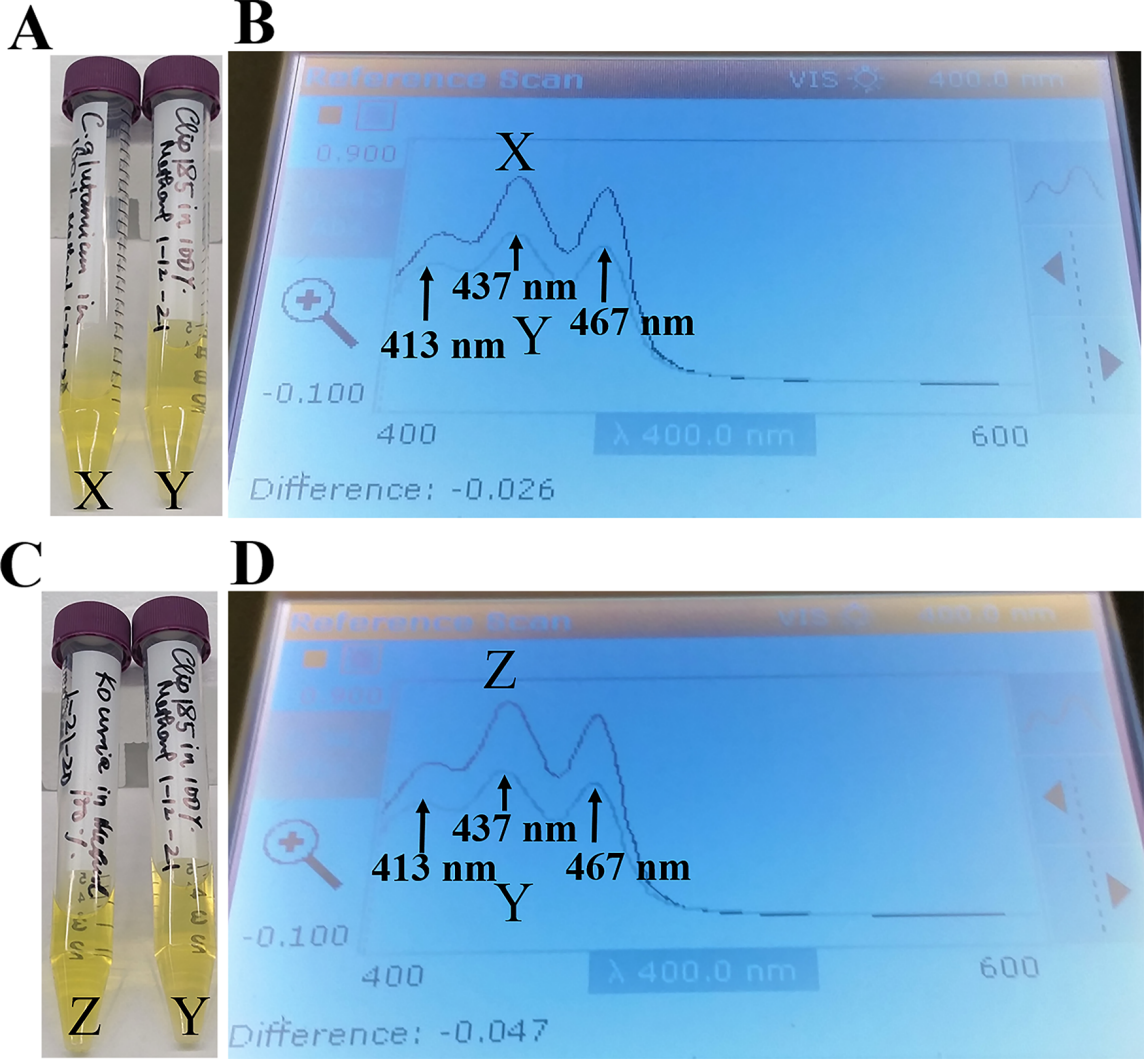
Spectrophotometric analyses of extracted pigments of Clip185. (A) Falcon tubes containing methanol-extracted pigments of *Corynebacterium glutamicum* (X) and Clip185 (Y). (B) Absorption spectra of extracted pigments *C. glutamicum* (X, dark blue spectrum) and Clip185 (Y, pale blue spectrum). (C) Falcon tubes containing methanol-extracted pigments of *Kocuria rhizophila* (Z) and Clip185 (Y). (D) Absorption spectra of extracted pigments *K. rhizophila* (Z, dark blue spectrum) and Clip185 (Y, pale blue spectrum). Three major absorption peaks of decaprenoxanthin are labeled.

Since our 16S rRNA partial gene sequencing showed that Clip185 is a *Microbacterium* sp. we extracted the yellow pigment from Clip185 ( Figure 12A; Figure 12C) and performed spectrophotometric analyses to determine whether Clip185 produces decaprenoxanthin ( Figure 12). As actinobacteria *C. glutamicum* and *Kocuria rhizophila* are known to produce decaprenoxanthin, ^16^^,^^30^ we used extracted pigments from these two bacterial strains ( Figure 12A; Figure 12C) as positive controls in the spectrophotometric assays ( Figure 12).

We found that the *C. glutamicum* (X spectrum in Figure 12B) and *K. rhizophila* (Z spectrum in Figure 12D) pigment absorption spectra exhibited three major absorption peaks that are representative of decaprenoxanthin absorption at 413 nm, 437 nm and 467 nm as described in the literature. ^30^^–^^32^ These three absorption peaks from *C. glutamicum* absorption spectrum X and *K. rhizophila* absorption spectrum Z very nicely overlaid against the three absorption peaks from the Clip185 absorption spectrum (Y spectrum in Figure 12B and Figure 12D). This result strongly suggests the presence of decaprenoxanthin in Clip185. HPLC and/or LC/MS analyses could confirm the presence of decaprenoxanthin in Clip185. However, we do not have access to such analytical tools at our institution.

### Light-regulation of decaprenoxanthin production in Clip185

**Figure 13.**
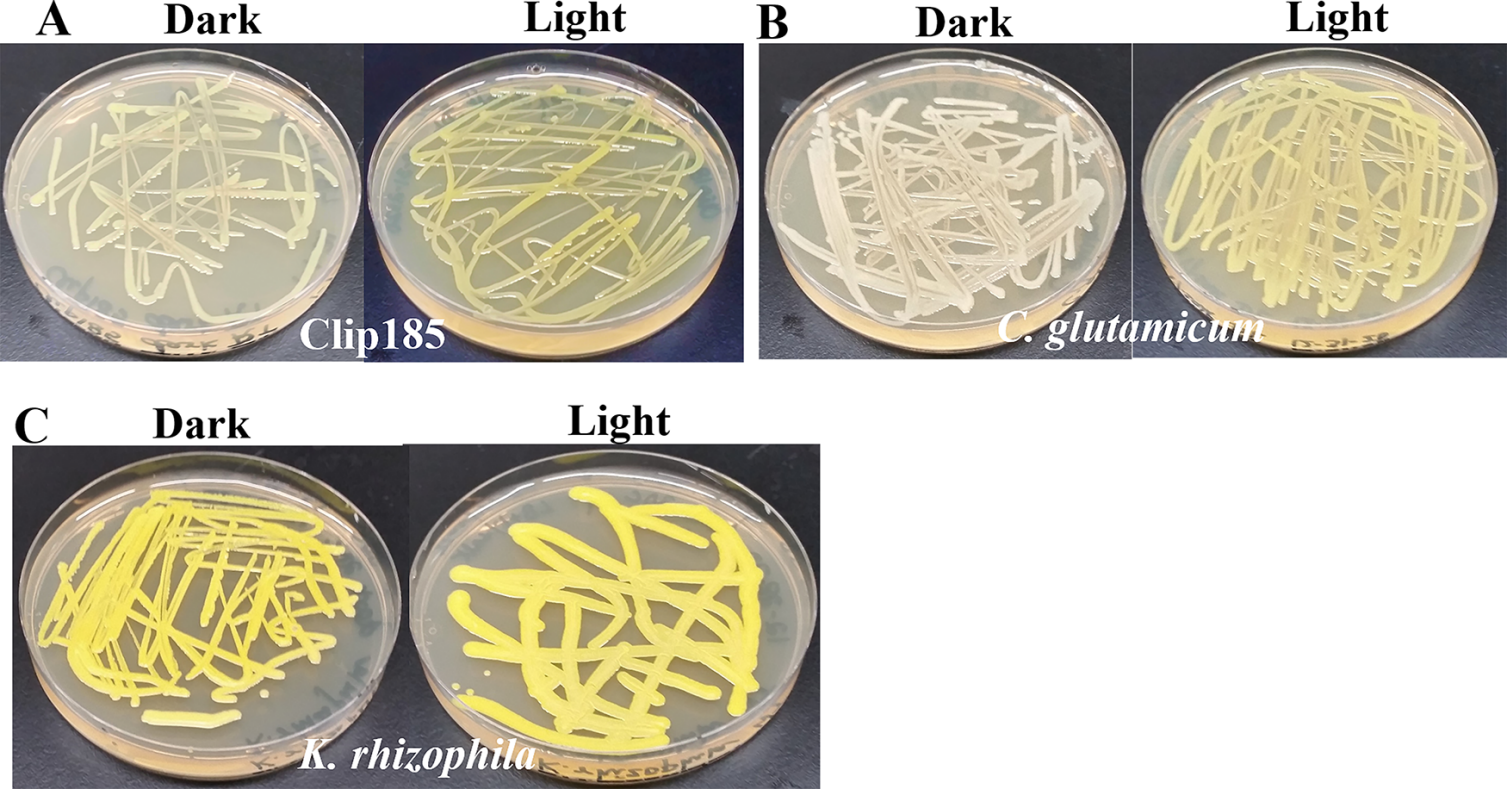
Decaprenoxanthin production under light and dark on LB media. (A) Decaprenoxanthin production in Clip185 under darkness and light. (B) Decaprenoxanthin production in *C. glutamicum* under darkness and light. (C) Decaprenoxanthin production in *K. rhizophila* under darkness and light. Bacterial culture plates were exposed to darkness or light for 5 days at 22°C and then imaged. Light intensity was 20-25 μmol m ^-2^s ^-1^.

It is known that in non-phototrophic bacteria, biosynthesis of C _50_ carotenoids can be modulated by growth conditions (e.g. presence/absence of light). ^15^^,^^46^^,^^47^ We studied the production of decaprenoxanthin in Clip185, *C. glutamicum* and *K. rhizophila* under dim light (20-25 μmol m ^-2^s ^-1^). In dark conditions, Clip185 appears cream color with a light yellowish tinge ( Figure 13A, left). Decaprenoxanthin production in Clip185 was induced under light ( Figure 13A, right). Decaprenoxanthin production is strongly induced under light in *C. glutamicum* as stated in published literature ^19^ in contrast to that in Clip185 ( Figure 13A; Figure 13B). *K. rhizophila* produces decaprenoxanthin constitutively irrespective of light conditions ( Figure 13C).

### Growth of Clip185 on LB media containing toxic concentrations of heavy metals

**Figure 14.**
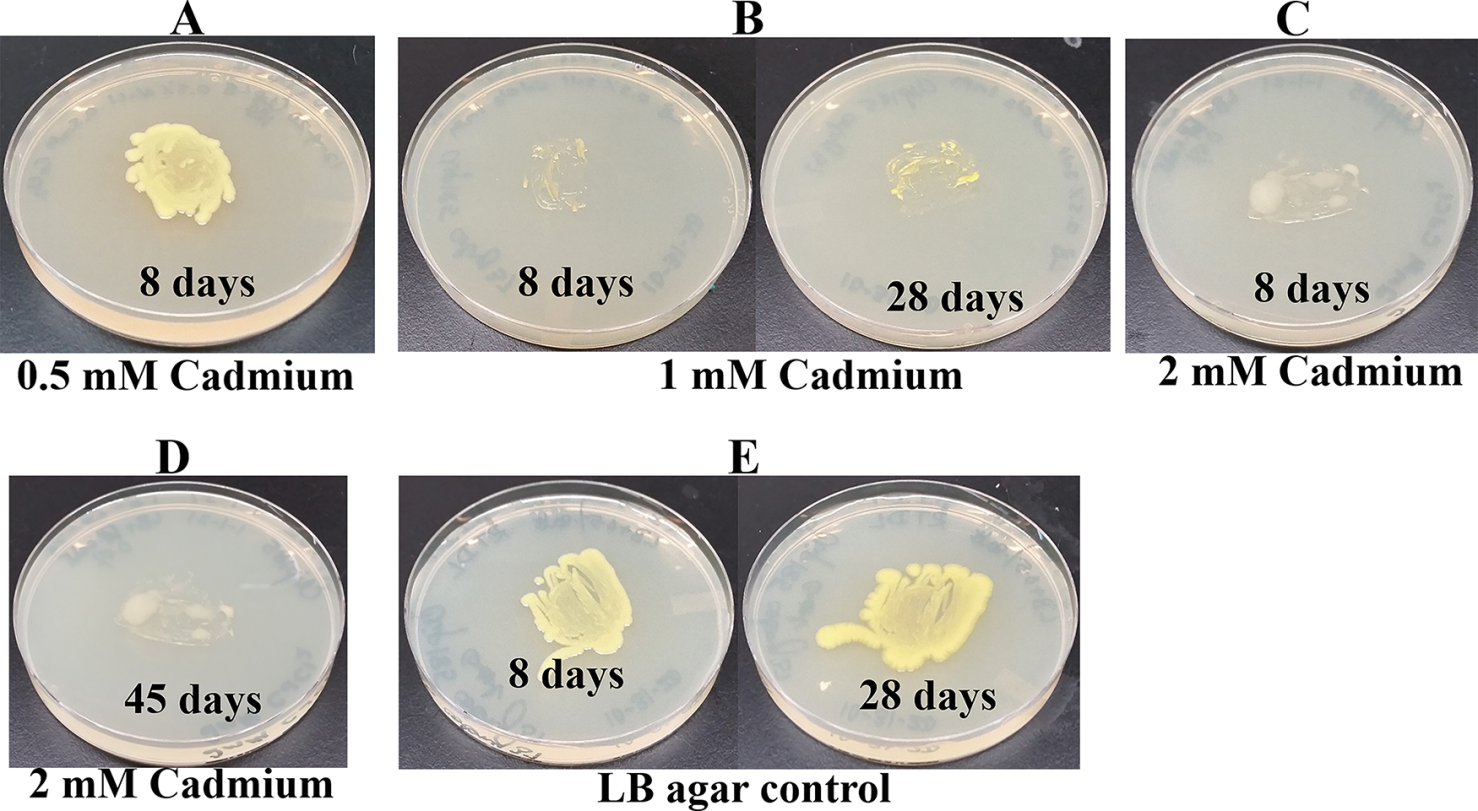
Growth of Clip185 on LB media containing Cadmium. (A) 8 days-growth of Clip185 on LB media containing 0.5 mM Cadmium. (B) 8 days (left) and 28 days (right)-growth of Clip185 on LB media containing 1 mM Cadmium. (C) 8 days-growth of Clip185 on LB media containing 2 mM Cadmium. (D) 45 days-growth of Clip185 on LB media containing 2 mM Cadmium. (E) 8 days (left) and 28 days (right)-growth of Clip185 on LB media (control). Number of days of growth are labeled on the figures. Light intensity was 10-15 μmol m ^-2^s ^-1^.

**Figure 15.**
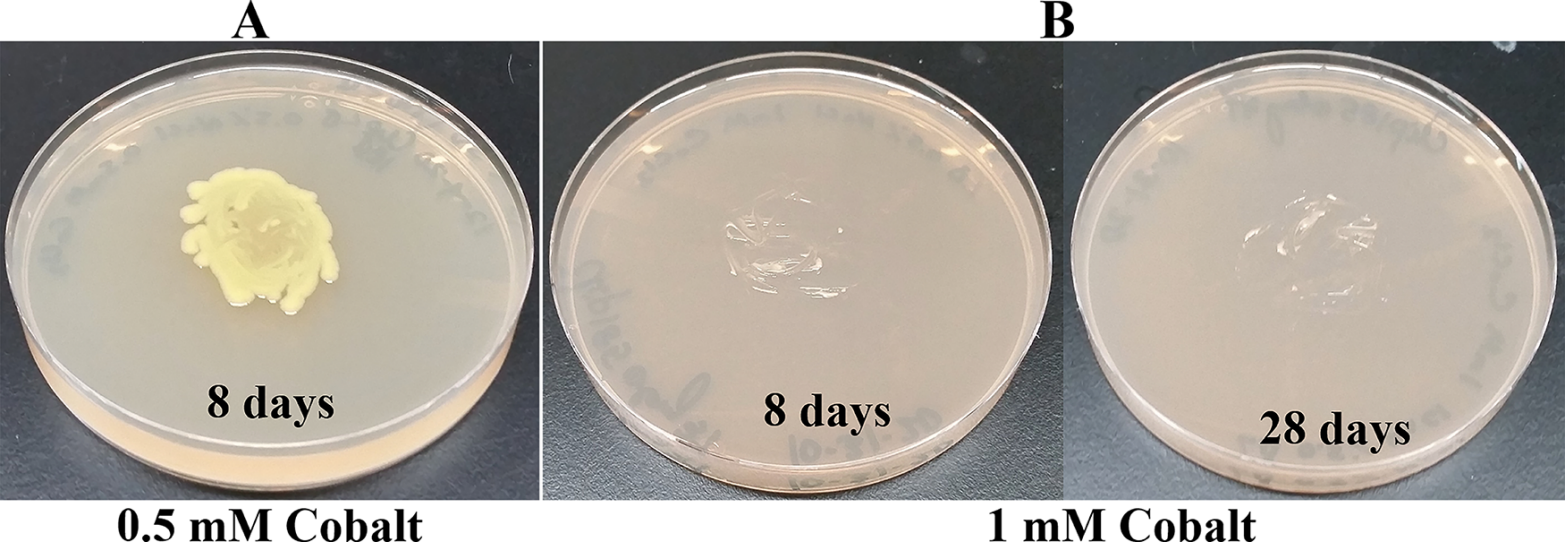
Growth of Clip185 on LB media containing Cobalt. (A) 8 days-growth of Clip185 on LB media containing 0.5 mM Cobalt. (B) 8 days (left) and 28 days (right)-growth of Clip185 on LB media containing 1 mM Cobalt. Number of days of growth are labeled on the figures. Light intensity was 10-15 μmol m ^-2^s ^-1^.

**Figure 16.**
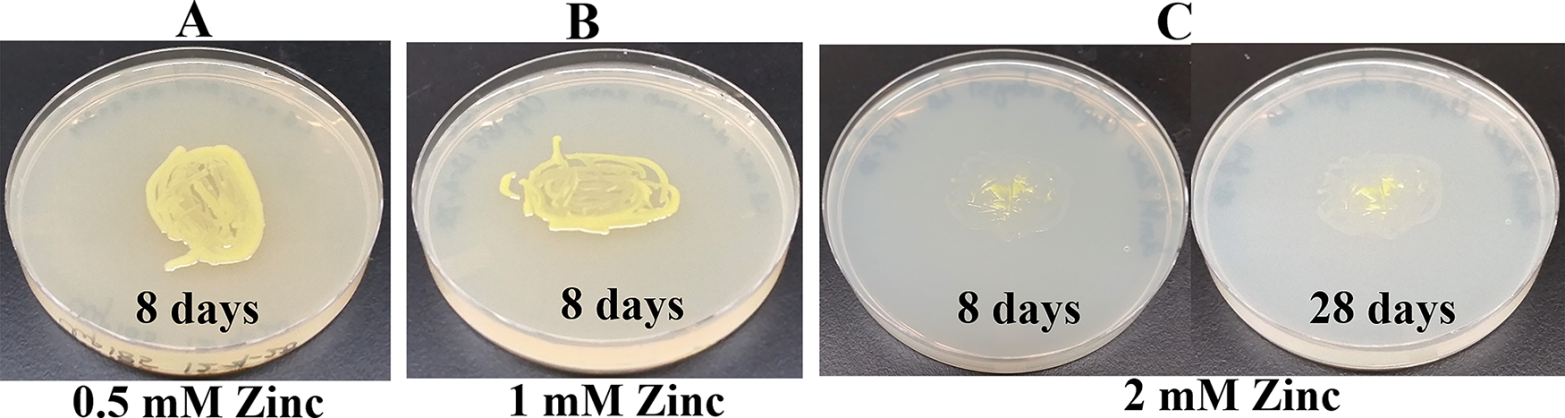
Growth of Clip185 on LB media containing Zinc. (A) 8 days-growth of Clip185 on LB media containing 0.5 mM Zinc. (B) 8 days-growth of Clip185 on LB media containing 1 mM Zinc. (C) 8 days (left) and 28 days (right)-growth of Clip185 on LB media containing 2 mM Zinc. Number of days of growth are labeled on the figures. Light intensity was 10-15 μmol m ^-2^s ^-1^. Light intensity was 10-15 μmol m ^-2^s ^-1^.

**Figure 17.**
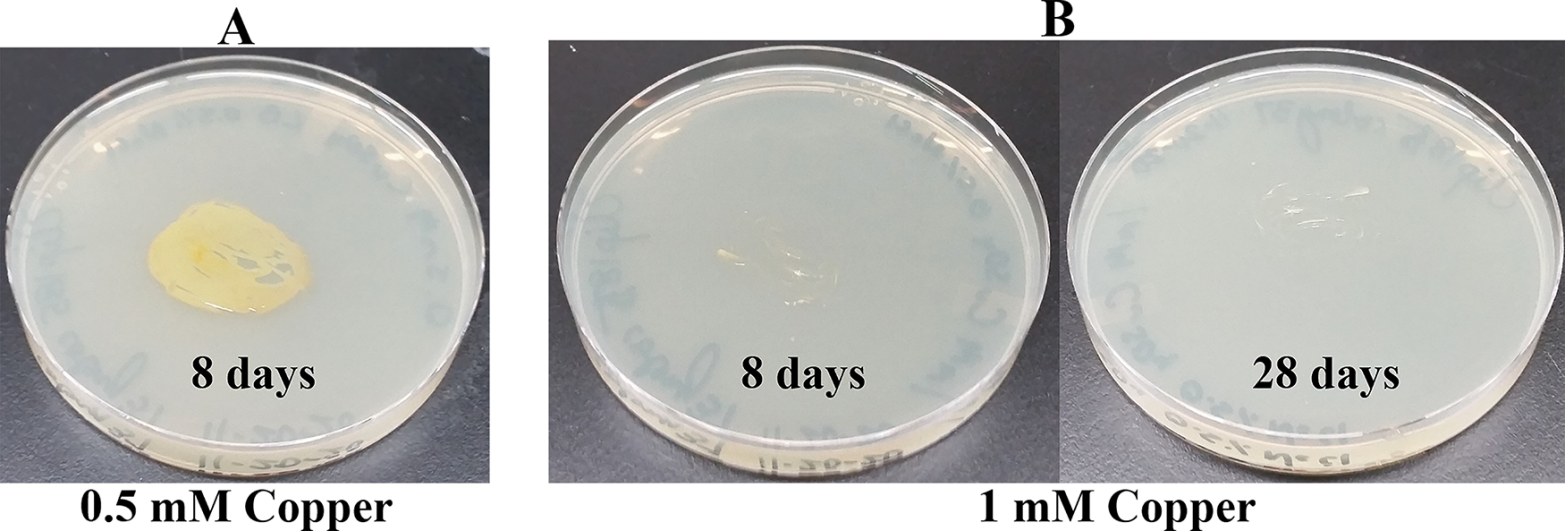
Growth of Clip185 on LB media containing Copper. (A) 8 days-growth of Clip185 on LB media containing 0.5 mM Copper. (B) 8 days (left) and 28 days (right)-growth of Clip185 on LB media containing 1 mM Copper. Number of days of growth are labeled on the figures. Light intensity was 10-15 μmol m ^-2^s ^-1^.

**Figure 18.**
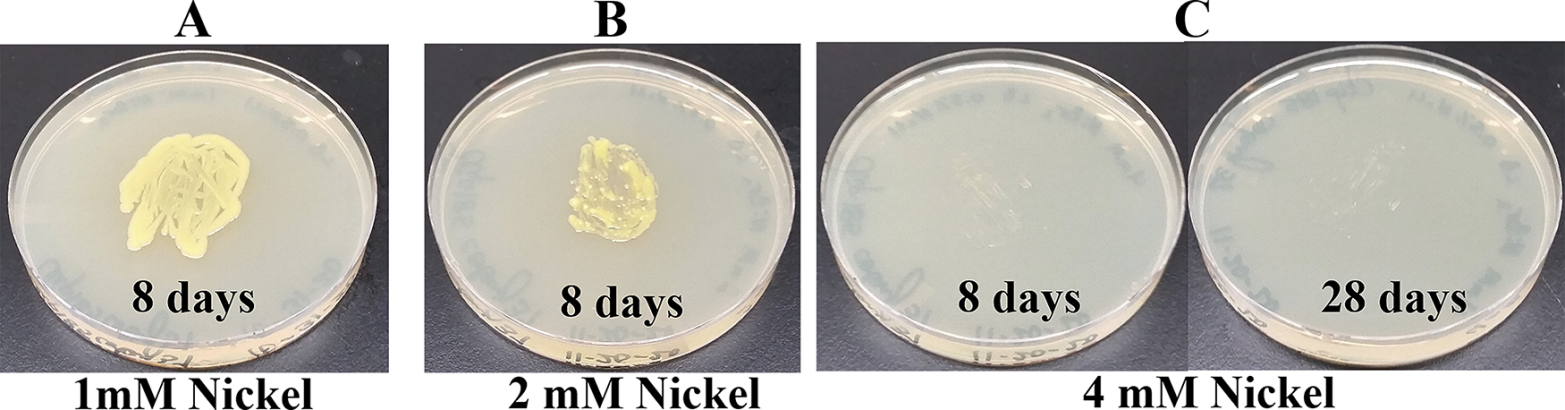
Growth of Clip185 on LB media containing Nickel. (A) 8 days-growth of Clip185 on LB media containing 1 mM Nickel. (B) 8 days-growth of Clip185 on LB media containing 2 mM Nickel. (C) 8 days (left) and 28 days (right)-growth of Clip185 on LB media containing 4 mM Nickel. Number of days of growth are labeled on the figures. Light intensity was 10-15 μmol m ^-2^s ^-1^.

**Figure 19.**
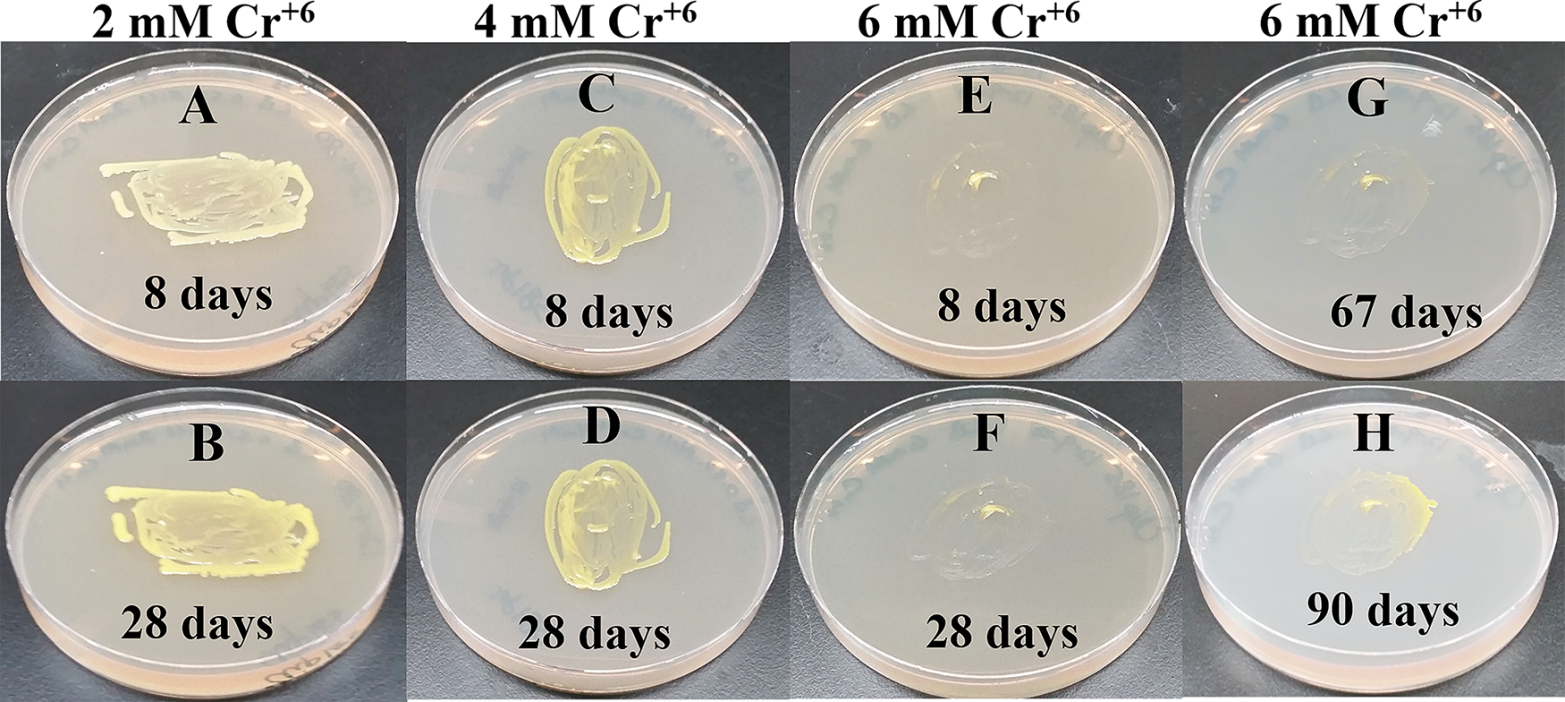
Growth of Clip185 on LB media containing hexavalent Chromium (Cr ^**6+**^). (A) 8 days-growth of Clip185 on LB media containing 2 mM Cr ^6+^. (B) 28 days-growth of Clip185 on LB media containing 2 mM Cr ^6+^. (C) 8 days-growth of Clip185 on LB media containing 4 mM Cr ^6+^. (D) 28 days-growth of Clip185 on LB media containing 4 mM Cr ^6+^. (E) 8 days-growth of Clip185 on LB media containing 6 mM Cr ^6+^. (F) 28 days-growth of Clip185 on LB media containing 6 mM Cr ^6+^. (G) 67 days-growth of Clip185 on LB media containing 6 mM Cr ^6+^. (H) 90 days-growth of Clip185 on LB media containing 6 mM Cr ^6+^. Number of days of growth are labeled on the figures. Light intensity was 10-15 μmol m ^-2^s ^-1^. See also Underlying data. ^48^

Since *Microbacterium sp.* has been isolated from heavy metal-contaminated environments, ^15^ we decided to test whether Clip185 could tolerate toxic concentrations (ranging from 0.5 mM - 6 mM) of six different heavy metals: cadmium, cobalt (Co ^2+^), zinc, copper (Cu ^2+^), nickel (Ni ^2+^) and chromium [Cr ^6+^] ( Figures 14- 19). Clip185 can grow well with yellow pigmentation on 0.5 mM of cadmium plate ( Figure 14A) but grows slowly on 1 mM cadmium plate and retains pigment production ( Figure 14B). Clip185 slowly grows on 2 mM cadmium plate, but over time stops growing and is white in color ( Figure 14C; Figure 14D). Growth of Clip185 for 8 days and 28 days on LB plate are shown for comparisons ( Figure 14E). Clip185 grew well with yellow pigmentation on 0.5 mM cobalt plate ( Figure 15A) but failed to grow on 1 mM cobalt plate ( Figure 15B). Clip185 grew well with yellow pigmentation on 0.5 mM and 1 mM zinc plates ( Figure 16A-B) but grew very slowly on 2 mM zinc plate ( Figure 16C). Clip185 grew well with yellow pigmentation on 0.5 mM copper plate ( Figure 17A) but failed to grow on 1 mM copper plate ( Figure 17B). Clip185 grew well with yellow pigmentation on 1 mM and 2 mM nickel plates ( Figure 18A-B) but failed to grow on 4 mM nickel plate ( Figure 18C). Clip185 could grow well with yellow pigmentation on 2 mM and 4 mM chromium plate ( Figure 19A-D) but grew extremely slowly with yellow pigmentation on 6 mM chromium plate ( Fig 19E-H). Clip185 failed to grow on 10 mM of chromium plate after 90 days of incubation (Underlying data ^48^).

## Discussion

Our biochemical and physiological analyses of Clip185 and its partial 16S rRNA gene sequencing have shown that Clip185 belongs to the *Microbacterium* taxon. *Microbacterium* belongs to the class Actinobacteria. Actinobacteria are mainly aerobic, gram-positive bacteria with a high G + C content and B-type cross linkages in peptidoglycan. ^49^^,^^50^ 124 species of *Microbacterium* have been identified with a validly published name in the List of Prokaryotic names with Standing in Nomenclature [LPSN] (last accessed 8-26-21). *Microbacterium* species have been isolated from a diverse range of environments and hosts. ^14^^,^^15^^,^^49^^,^^51^^–^^53^ We isolated Clip185 from a contaminated TAP medium plate of *C. reinhardtii* but, it surprisingly did not grow well on TAP agar (not enriched in amino acids/peptides) like it grew on LB agar, which is enriched in peptides, amino acids and vitamins ( Figure 1; Figure 4). It also did not grow well on PDA (not enriched in amino acids/peptides) while it grew fine on MHA, which contains beef extract and acid hydrolysate of casein as rich source of amino acids (Underlying data ^37^). These results taken together indicate that Clip185 has a high amino acid/peptide/vitamin requirement for growth and is a fastidious bacterium.

Clip185 was resistant to penicillin, chloramphenicol, neomycin, and polymyxin B but was very sensitive to the cell wall-disrupting antibiotic vancomycin ( Table 1, Underlying data; ^35^ Figure 2). Thirteen *Microbacterium* isolates from metal-contaminated soil in Seymour, Indiana (USA) were resistant to chloramphenicol, ampicillin and vancomycin. ^27^ In contrast, *M. yannicii* PS01, a multi-drug resistant strain isolated from a cystic fibrosis patient was sensitive to chloramphenicol, amoxicillin, vancomycin and colistin (polymyxin E) but resistant to Tobramycin ^49^ (an aminoglycoside antibiotic like neomycin, that binds to the 30S subunit of the prokaryotic ribosome).

Bacteremia-causing *Microbacterium sp.* have been isolated from clinical specimens like catheter, blood etc. ^54^^–^^56^ Partial 16S rRNA gene of Clip185 has a 99.1% sequence identity with that of *M. binotii* PK1-12M strain whose genome has not been sequenced yet ( Figure 10B). There was a reported case of bacteremia caused by *M. binotii* in a sickle cell anemia patient. ^56^ *M. binotii* strains CIP 101303T and CIP 102116 from the Collection de l’Institut Pasteur were isolated from human blood in 1976 and 1977, respectively. ^57^ As Clip185 is resistant to four major antibiotics, it has the potential to become an opportunistic human pathogen as previously reported for other species of *Microbacterium*, especially in immune-compromised patients.

Several heavy metal-tolerant environmental isolates of *Microbacterium* exist in the literature. ^15^^,^^20^^–^^26^ One of the two nearest relatives of Clip185, *M. binotii* strain PK1-12M, was isolated from primary peat swamp forest soil in the Suratthani Province of Thailand (Accession number: MN428150.1). Peat soils are known to be enriched in heavy metals, especially in raised bogs located near mining and smelting areas. ^58^ Lowland rain forests and salt-water mangrove forests typically surround peat swamp forests near the coast. ^59^ Four *Microbacterium* bio-emulsifier-producing strains were isolated from oil contaminated mangrove sediments in Guanabara Bay (Rio de Janeiro, Brazil). These strains were able to remove cadmium and zinc from contaminated industrial sites with varying abilities according to the carbon sources used. ^59^ In 2021, BLAST analyses identified *Microbacterium sp.* strain MDP6 as the nearest relative of Clip185 (Accession number: MK128451.1). *Microbacterium sp.* strain MDP6 strain is a zinc- and cadmium-tolerant endophytic bacteria isolated in Thailand from *Murdannia spectabilis* (Kurz) Faden. with plant growth-stimulating properties. ^60^

To the best of our knowledge we could not find reports in the literature about the minimum concentration of different heavy metals tolerated by bacteria. Heavy metal-tolerance of sixteen different strains of *Microbacterium* sp. isolated from metal-contaminated soil samples in Seymour, IN, USA, was studied by a research group. ^27^ 11 of the 16 *Microbacterium* isolates were able to tolerate concentrations (0.1 mM) of cobalt, cadmium, and nickel, in addition toand 0.5 mM–20 mM Cr(VI) (0.5–20 mM). Four of these 16 *Microbacterium* isolates displayed lack of cadmium tolerance. ^27^ Clip 185 can tolerate 2 mM of cadmium, zinc & nickel, 0.5 mM of cobalt and copper and 6 mM of Cr ^6+^ on LB-agar ( Figures 14- 19). Clip185 showed a higher cadmium, nickel and zinc tolerance but lower Cr ^6+^ tolerance than the isolated 16 *Microbacterium* strains state above. ^27^ Based on Clip185’s heavy-metal tolerance we can speculate that it can thrive in heavy-metal enriched ecological niches in nature. We used LB in our lab to grow Clip185. LB is a nutrient-rich medium that allows growth of most bacteria. Fastidious bacteria have complex nutritional requirements. Literature reviews have shown that researchers usually use Tryptic Soy Agar/Broth (TSA/B) to grow *Microbacterium.* ^15, 16^ TSA/B is a complex nutrient medium that has a higher concentration of amino acids/peptides and carbohydrates compared to that in LB. Hence it is possible that Clip185 has a higher heavy-metal tolerance capability which was not on full display when grown on the LB media used in our experiments. In the future, we would like to assess the heavy-metal tolerance of Clip185 in TSA/B more fully.

Extracellular heavy metal barriers, extracellular metal sequestration, active efflux of metal ions, intracellular metal sequestration, and reduction of metal ions are some of the microbial protective mechanisms employed to combat heavy metal-induced stress. ^61^^–^^63^ In the future, we want to spectrophotometrically monitor the growth of Clip185 at 600 nm in TSB containing heavy metals, to determine if Clip185 is able to resist heavy metals. ^16^^,^^27^ In the environment, Cr ^6+^, (a soluble, highly toxic form of chromium) is reduced to Cr ^3+^, an insoluble, less toxic form. ^64^ Clip185’s ability to reduce Cr ^6+^ to Cr ^3+^ in TSB can be tested using spectrophotometric assays as described in Henson *et al.*, 2015. ^16^ Results from these experiments will help us to conclusively determine the metal-mobilizing ability of Clip185, and possibly its potential use in bioremediation.

Carotenoids are yellow-red-colored pigments found in all photosynthetic eukaryotes and prokaryotes as well as in non-phototrophic microbes. ^65^ Prokaryotic carotenoids display diverse chemical structures compared to the eukaryotic ones. ^66^ Carotenoids have diverse biological roles (e.g. as anti-oxidants and light harvesting pigments, photo-protection, acting as membrane stabilizers or repressors of translational surveillance and defense pathways in nematodes etc.). ^30,^ ^67^^,^^68^ Commercially, carotenoids are used as food colorants, animal feed supplements, cosmetics, antioxidants and other health supplements. ^67^^,^^69^ Pathways and related enzymes for carotenoid biosynthesis are well characterized ( Figure 11).

C _40_ carotenoids are the predominant class of carotenoids. C _30_ and C _50_ carotenoids are rare. ^70^ To date C _50_ carotenoids have mainly been isolated from non-phototrophic bacteria like the halophilic Archaebacteria, *Halobacteria* and *Halococcus*, ^71, 72^ gram-positive Actinobacteria ^18^ and one gram-negative bacterium, *Pseudomonas.* ^16, 73^ C _50_ carotenoids have multiple conjugated double bonds and polar hydroxyl groups, which influences their anti-oxidant and light harvesting properties. These chemical properties make them attractive candidates for their use in photo-protective cosmetics and sun screens. ^16^^,^^18^

Whole genome sequencing of *Microbacterium* sp. strain PAMC28756, isolated from *Stereocaulon sp.*, an Antarctic lichen, revealed the presence of C _50_ carotenoid biosynthesis gene clusters. ^74^ Recently, C _50_ carotenoid biosynthesis gene clusters were shown to be the most common secondary metabolite gene clusters in 70 *Microbacterium* strains isolated from metal contaminated and non-contaminated sites. ^15^ We have shown that Clip185 produces decaprenoxanthin like actinobacteria, *C. glutamicum and K. rhizophila* ( Figure 12). In non-phototrophic bacteria, carotenoid production is mainly classified into three types: 1) light-inducible, 2) constitutive, and 3) cryptic. ^66^^,^^75^ A majority of bacteria produce carotenoids constitutively but, bacteria like *Myxococcus*, *Streptomyces*, *Mycobacterium*, *Agromyces* and *Sulfolobus* only produce carotenoids under light. Carotenoid production in two *Streptomyces* species, i.e. *Streptomyces setonii* and *Streptomyces griseus* are designated as cryptic, since the growth conditions that trigger carotenoid biosynthesis in these bacteria are unknown at this point. ^66^^,^^75^ Carotenoid production is weakly induced in two environmental isolates of *Microbacterium*, i.e. *M. phyllosphaerae* and *M. foliorum* but strongly induced in two isolates of *M. natoriense.* ^19^

We have shown that Clip185 synthesizes decaprenoxanthin under light, but the induction is not as strong as that seen in *C. glutamicum* ( Figure 13A-B). In 2019, Sumi *et al.* ^19^ have shown that light strongly induces decaprenoxanthin production in *C. glutamicum*, and our findings consolidate this ( Figure 13B). *K. rhizophila* produces decaprenoxanthin irrespective of light conditions ( Figure 13C). Both Clip185/ *Microbacterium* and *Kocuria* belong to the suborder of Micrococcineae under the order Actinomycetales. This shows that the regulation of decaprenoxanthin production in closely related Actinobacteria can differ. CrtR is a transcriptional regulator in the MarR family that represses crtE gene ( Figure 11) transcription in the dark in Micrococcales and Corynebacteriales members. ^19^ It would be interesting to check whether the CrtR gene is present and/or altered in Clip185 compared to that in *C. glutamicum* ^19^ ( Figure 13A-B) once we have sequenced the whole genome of Clip185.

Secondary metabolism occurs in bacteria during stationary growth phases, when the energy and carbon pool is diverted away from biomass production towards the production of secondary metabolites like antibiotics, carotenoids etc. ^76^ Pigment production can be modulated by changing the carbon and nitrogen sources and salt concentrations in the growth medium. ^77^ In the future, we would like to test different carbon and nitrogen sources and salt concentrations to see if we can formulate a growth medium that will enhance decaprenoxanthin production in the stationary phase.

Carotenoids help to quench reactive oxygen species (ROS) which are generated under abiotic, stressful conditions. To the best of our knowledge, very little information is available about functional roles of decaprenoxanthin in combating specific types of abiotic stresses. Clip185 growth can be monitored spectrophotometrically in an optimized growth medium under different ROS-generating stressful conditions (e.g. high light, in the presence of ROS like hydrogen peroxide or single oxygen generators like Rose Bengal; UVB light; salt and temperature stress, heavy metal stress etc.). The results from these experiments can shed light on specific stresses that stimulate decaprenoxanthin biosynthesis in bacteria, and might have applications in improving decaprenoxanthin production on a commercial scale.

Currently the NCBI genomic database has 293 *Microbacterium* genome assemblies from environmental isolates without a validly published species name (last accessed on 8-26-2021). Clip185’s nearest relatives are two *Microbacterium* strains, whose whole genome sequencing data are not available on NCBI at the time of writing. The *Microbacterium* genus contains species that have highly similar 16S rRNA gene sequences and are difficult to identify at the species level, simply based on 16S rRNA gene sequences. ^13^

Because of funding limitations at the time of submission of this manuscript, we could not sequence the whole genome of Clip185. We will receive funding in fall 2021 for whole Clip185 genome sequencing. Clip185 displayed variations in antibiotic sensitivity and heavy metal tolerance when compared to other *Microbacterium* isolates from metal-contaminated soil and a clinical setting. ^27^^,^^49^ These observed variations can stem from the differences among specific ecological niches from where these bacterial strains were isolated. Comparative whole genome sequence analyses of different environmental *Microbacterium* isolates with that of Clip185 will show us the importance of an ecological niche in shaping genetic diversity in a species, in addition to clarifying its taxonomic identity.

## Data availability

### Underlying data

NRRL ARS Culture Collection: Clip185/ *Microbacterium binotii* strain PK1-12M variant; Accession number: B-65609.

NCBI GenBank: *Microbacterium binotii* strain PK1-12M variant; 16S ribosomal RNA gene, partial sequence, Accession number MN633284.1: https://www.ncbi.nlm.nih.gov/nuccore/MN633284.1.

Figshare: Antibiotic sensitivity data of the bacterial strain Clip185/ *Microbacterium binotii* strain PK1-12M variant (Accession number: MN633284.1) and *Chlamydomonas reinhardtii* strain 4A+ from disc diffusion antibiotic susceptibility tests, https://doi.org/10.6084/m9.figshare.14668914. ^35^

This project contains the following underlying data:

• Supplemental Data S1 (XLSX). Contains means and standard deviations of zones of growth inhibitions of Clip185 and *Chlamydomonas* on antibiotic containing TAP plates.
• Supplemental Data S2 (XLSX). Statistical analyses of the zones of growth inhibitions of the bacterial strain Clip185 and *Chlamydomonas* induced by five antibiotics.

Figshare: Image of a LB plate with single colonies of Clip185/ *Microbacterium binotii* strain PK1-12M variant (Accession number: MN633284.1), https://doi.org/10.6084/m9.figshare.14669214. ^34^

This project contains single colonies of Clip185 Clip185/ *Microbacterium binotii* strain PK1-12M variant (Accession number: MN633284.1) on a LB plate.

Figshare: Images of Clip185/ *Microbacterium binotii* strain PK1-12M variant (Accession number: MN633284.1) growth on Potato Dextrose Agar (PDA) and Mueller-Hinton Agar (MHA), https://doi.org/10.6084/m9.figshare.14669397. ^37^

This project contains an image that shows the growth of Clip185 on Potato Dextrose Agar (PDA) and Mueller-Hinton Agar (MHA).

Figshare: Image of the DNA agarose gel showing the electrophoretic analysis of PCR products obtained using the 16S rRNA gene-specific primers on Clip185/ *Microbacterium binotii* strain PK1-12M variant (Accession number: MN633284.1) genomic DNA, https://doi.org/10.6084/m9.figshare.14669400. ^42^

This project contains one DNA agarose gel image that shows DNA agarose gel electrophoresis analysis of PCR products obtained using the 16S rRNA gene-specific primers on Clip185/ *Microbacterium binotii* strain PK1-12M variant (Accession number: MN633284.1) genomic DNA.

Figshare: Image of growth of Clip185/ *Microbacterium binotii* strain PK1-12M variant (Accession number: MN633284.1) on a LB plate containing 10 mM hexavalent Chromium (Cr ^6+^), https://doi.org/10.6084/m9.figshare.14669421. ^48^

This project contains one image of a 10 mM hexavalent Chromium (Cr ^6+^) containing LB media plate on which Clip185/ *Microbacterium binotii* strain PK1-12M variant (Accession number: MN633284.1) was streaked and incubated for 90 days for growth analysis.

Data are available under the terms of the Creative Commons Attribution 4.0 International license (CC-BY 4.0).
