# Supplementary material for: Isolation and characterization of a heavy metal- and antibiotic-tolerant novel bacterial strain from a contaminated culture plate of Chlamydomonas reinhardtii, a green micro-alga
Source: F1000Res. 2021 Sep 7;10:533. Originally published 2021 Jul 5. [Version 2] doi: 10.12688/f1000research.53779.2 (PMC8424464; doi:10.12688/f1000research.53779.2)
Supplement: Peer review history

# Peer review: Reviewer response for version 2

**DOI:** 10.5256/f1000research.77092.r93628

**Published:** 2021-09-13

**Authors:** Ruby A. Ynalvez

**Affiliations:** 1 Department of Biology and Chemistry, Texas A&M International University, Laredo, TX, USA

The authors have addressed all my queries and have incorporated my recommendations. I no longer have any comments nor suggestions.

Is the work clearly and accurately presented and does it cite the current literature?

Yes

If applicable, is the statistical analysis and its interpretation appropriate?

I cannot comment. A qualified statistician is required.

Are all the source data underlying the results available to ensure full reproducibility?

Yes

Is the study design appropriate and is the work technically sound?

Yes

Are the conclusions drawn adequately supported by the results?

Yes

Are sufficient details of methods and analysis provided to allow replication by others?

Yes

Reviewer Expertise:

Cell biology and biochemistry

I confirm that I have read this submission and believe that I have an appropriate level of expertise to confirm that it is of an acceptable scientific standard.

# Peer review: Reviewer response for version 2

**DOI:** 10.5256/f1000research.77092.r93629

**Published:** 2021-09-07

**Authors:** David Dewez

**Affiliations:** 1 Laboratory of Environmental and Analytical Biochemistry of Contaminants, Department of Chemistry, University of Quebec at Montreal, Montreal, QC, Canada

The authors made all the corrections suggested. I do not have any new comments.

Is the work clearly and accurately presented and does it cite the current literature?

Yes

If applicable, is the statistical analysis and its interpretation appropriate?

Yes

Are all the source data underlying the results available to ensure full reproducibility?

Yes

Is the study design appropriate and is the work technically sound?

Yes

Are the conclusions drawn adequately supported by the results?

Yes

Are sufficient details of methods and analysis provided to allow replication by others?

Yes

Reviewer Expertise:

Toxicology and chemistry of aquatic microorganisms.

I confirm that I have read this submission and believe that I have an appropriate level of expertise to confirm that it is of an acceptable scientific standard.

# Peer review: Reviewer response for version 1

**DOI:** 10.5256/f1000research.57195.r89082

**Published:** 2021-08-12

**Authors:** Ruby A. Ynalvez

**Affiliations:** 1 Department of Biology and Chemistry, Texas A&M International University, Laredo, TX, USA

The title of the article under review is appropriate for the content of the article. In addition, the abstract represents a suitable and complete summary of this interesting work. Most current literature was cited in the article. The authors were able to clearly present their work on the isolation, taxonomic identification, and physiological and biochemical characterizations of Clip185. Using several microbiological tests, the authors were able to characterize Clip 185. Their disc diffusion assays showed that Clip 185 is very sensitive to vancomycin but resistant to penicillin and other bacterial cell membrane- and protein synthesis-disrupting antibiotics. In addition, the results of their experiment suggests the potential use of vancomycin in preventing not only *Microbacterium* species but also other bacteria without being detrimental to *C. reinhardtii*.

 Results also showed that Clip185 is likely to produce a C50 carotenoid, decaprenoxanthin, which is a powerful anti-oxidant with commercial demand. A confirmation of decaprenoxanthin via analytical tools, i.e. HPLC, is recommended for future studies. Clip185 is able to tolerate toxic concentrations of six heavy metals. This finding is relevant in future bioremediation studies. NCBI-BLAST analyses of the partial 16S rRNA gene sequence of Clip185 revealed a 99% sequence identity to that of *Microbacterium binotii *strain PK1-12M and *Microbacterium sp. *strain MDP6. Interestingly, Clip185 could be a potentially new strain of *Microbacterium binotii* or a new *Microbacterium *species. Overall, this is an interesting article. The findings of their research will open up new research ideas related to bioremediation, antioxidants, gene isolation and regulation, and biotechnology. Thus, the publication of this article will warrant the attention and interest of scientists from diverse fields. I only have several comments that the authors need to consider:

 In Keywords:

- Italicize "Chlamydomonas", "Microbacterium binotii".

In Introduction:

- Tris-Phosphate-Acetate (TAP) change to Tris- Acetate- Phosphate (TAP).
- It was mentioned that Clip 185 grows better in a Lysogeny Broth (LB) medium than in a TAP medium and although it was mentioned later in the article, I think it will be to the benefit of the readers to right away mention why the bacterium grows better in LB.
- Clip185 could grow in the presence of 6mM chromium, 2mM of nickel, cadmium and zinc, and 0.5 mM of copper and cobalt in the LB medium. As a reader, I was wondering what will be the bacteria’s minimum tolerable concentration of each of these heavy metals? It will be helpful to have these tolerable concentrations as reported in the literature. It does not have to be *Microbacterium* *sp.,* but if there were reports for *Microbacterium sp*. then the better.

In Methods:

- Antibiotic plates were incubated at 22°C. Clip185 and *Chlamydomonas *plates were imaged after 7 days of incubation. On the other hand, streaked TAP + vancomycin plates were imaged after incubation at 22°C for 2.5 weeks. What is the reason there is a difference in incubation, 7 days versus 2.5 weeks between the other antibiotics and that of vancomycin?
- In Clip 185 growth analysis: The same thing, it will be clearer to all readers if the authors will add why LB-agar and TAP agar culture plates were imaged after 3 days and 7 days of growth, respectively.
- The authors specified why *S. aureus* was used in MSA. Thus, also specify why *Acidovorax sp *was used on TSBA.
- Please indicate why *Corynebacterium glutamicum* strain ATCC13032 and *Kocuria rhizophila* ATCC?? were used along with Clip 185 testing light-regulation of decaprenoxanthin production? Although it was mentioned later in the article why, I think it’s better to clarify this already in the Methods section, again, just like how it was clear why *S. aureus* was used in MSA.
- It was mentioned that there were three replicates per metal concentration in the growth assays on LB media containing heavy metals experiment. On the other hand, how many trials were there per replicate? Same thing, three replicates per strain were used in the decaprenoxanthin extractions and spectrophotometric analyses experiment. On the other hand, how many trials were there per replicate?
- There was no mention of how many replicates and trials were performed in the other experiments. I recommend mentioning the number of replicates and trials within replicates for the other experiments.

In Results:

- Grey and white rows represent 50 µg and 100 µg of each antibiotic applied on the filter paper discs – please double-check Table 1.
- Authors stated: “We used polymyxin B amounts that were 1.4- fold to 2.8-fold higher than what is used for KB tests, and growth inhibition zones of *Chlamydomonas* and Clip185 were less than 13 mm (Table 1).” Why not use less than 12 mm instead of less than 13 mm to be consistent with a previous statement that used less than 12 mm as a reference?
- In Figure 9, it could be helpful to add: Both Clip185 (B) and (D) were tested positive in the starch hydrolysis test as shown by a visible clear zone indicated by the arrows surrounding the growth (or if not an arrow perhaps encircle that area, so that the clear zone will be more easily seen by the readers).
- I recommend a revision to: This result strongly suggests the presence of decaprenoxanthin in Clip185. HPLC and/or LC/MS analyses could confirm the presence of decaprenoxanthin in Clip185. However, we do not have access to such analytical tools at our institution.
   
  - Original statements were: This result very strongly indicates the presence of decaprenoxanthin in Clip185. We do not have access to such analytical tools at our institution.
- Please double-check the figures and the corresponding figure titles for Figures 15, 16, and 17. For example, Figure 15 has plates labelled Zinc but the title is Figure 15. Growth of Clip185 on LB media containing Cobalt.

Thank you for the opportunity to review this article.

Is the work clearly and accurately presented and does it cite the current literature?

Yes

If applicable, is the statistical analysis and its interpretation appropriate?

I cannot comment. A qualified statistician is required.

Are all the source data underlying the results available to ensure full reproducibility?

Yes

Is the study design appropriate and is the work technically sound?

Yes

Are the conclusions drawn adequately supported by the results?

Yes

Are sufficient details of methods and analysis provided to allow replication by others?

Yes

Reviewer Expertise:

Cell biology and biochemistry

I confirm that I have read this submission and believe that I have an appropriate level of expertise to confirm that it is of an acceptable scientific standard.

# Response

**Published:** 2021-08-26

**Authors:** Mautusi Mitra

**Affiliations:** University of West Georgia, USA

Please see our point-by-point responses to your queries given below. Thanks for investing your time and energy to review our manuscript and providing us valuable inputs to improve the manuscript quality. Hope you will be willing to review our version 2 of the article that has addressed your comments.

**1. Reviewer’s query/comments for Keywords: **Italicize "Chlamydomonas", "Microbacterium binotii".

**Our answer:** We had the above-stated two words in italics when we submitted our article in version 1. The italicization was not retained after publication as the italics function is not yet implemented into this section of the F1000Research article management system. Hence we are unable to implement this change.

**2. Reviewer’s query/comments for Introduction section:**

 a. **Reviewer’s query/comments:**  Tris-Phosphate-Acetate (TAP) change to Tris- Acetate- Phosphate (TAP).

**Our answer:** We have made the change.

 b. **Reviewer’s query/comments:** Clip185 could grow in the presence of 6 mM chromium, 2 mM of nickel, cadmium and zinc, and 0.5 mM of copper and cobalt in the LB medium. As a reader, I was wondering what will be the bacteria’s minimum tolerable concentration of each of these heavy metals? It will be helpful to have these tolerable concentrations as reported in the literature. It does not have to be Microbacterium sp., but if there were reports for Microbacterium sp. then the better.

**Our answer: **We agree that this is a very good suggestion. We could not find any literature record of minimum concentrations of different heavy metals tolerated by *Microbacterium sp.* or by any species of bacteria. There is a report of heavy metal tolerance of 16 *Microbacterium sp.* strains isolated from metal-contaminated soil in Indiana, USA. We have compared the heavy metal tolerance of Clip185 with these 16 *Microbacterium sp.* isolates and have added the information with citations under Introduction and also in paragraph 5 under the Discussion section.

 3. **Reviewer’s query/comments for the** **Methods section:**

 a. **Reviewer’s query/comments**: Antibiotic plates were incubated at 22°C. Clip185 and *Chlamydomonas* plates were imaged after 7 days of incubation. On the other hand, streaked TAP + vancomycin plates were imaged after incubation at 22°C for 2.5 weeks. What is the reason there is a difference in incubation, 7 days versus 2.5 weeks between the other antibiotics and that of vancomycin?

**Our answer:** We have added the explanation for monitoring Clip185 growth for 3 days on LB plates vs. monitoring Clip185 growth for 7 days on TAP plates under the Methods section sub-section: Testing antibiotic sensitivity of Clip185. We have also revised the Results section sub-section: Clip185 antibiotic susceptibility tests accordingly. We wanted to see if the selected amount of vancomycin was bactericidal or bacteriostatic. In other words, we wanted to see if we will see re-growth of Clip185 on the antibiotic-containing plate if we have a longer incubation of 2.5 weeks compared to 7-days incubation.

 b. **Reviewer’s query/comments**: In Clip 185 growth analysis: The same thing, it will be clearer to all readers if the authors will add why LB-agar and TAP agar culture plates were imaged after 3 days and 7 days of growth, respectively.

**Our answer: ** We have added the explanation in the Methods section sub-section: Clip185 growth analyses and also have revised the result section sub-section: Clip185 growth analyses on TAP- and LB-agar medium plates at different temperatures.

 c. **Reviewer’s query/comments**: The authors specified why *S. aureus* was used in MSA. Thus, also specify why *Acidovorax sp.* was used on TSBA.

**Our answer:** We have added the explanation under the Methods section sub-section: Growth assays on Tryptic Soy Blood Agar (TSBA) and also have revised the Results section sub-section: Testing for Clip185’s ability to lyse red blood cells.

 d. **Reviewer’s query/comments**: Please indicate why *Corynebacterium glutamicum* strain ATCC13032 and *Kocuria rhizophila* ATCC?? were used along with Clip 185 testing light-regulation of decaprenoxanthin production? Although it was mentioned later in the article why, I think it’s better to clarify this already in the Methods section, again, just like how it was clear why *S. aureus* was used in MSA.

**Our answer: **We have added the explanation under the Methods section sub-section: Decaprenoxanthin extractions and spectrophotometric analyses. We also added the *K. rhizophila* ATCC strain number (ATCC 9341) in version 2.

 e. **Reviewer’s query/comments**: It was mentioned that there were three replicates per metal concentration in the growth assays on LB media containing heavy metals experiment. On the other hand, how many trials were there per replicate? Same thing, three replicates per strain were used in the decaprenoxanthin extractions and spectrophotometric analyses experiment. On the other hand, how many trials were there per replicate?

**Our answer: **We had three biological replicates. Each biological replicate had three internal replicates. This information has been added under the Methods section sub-sections: Growth assays on LB media containing heavy metals and Decaprenoxanthin extractions and spectrophotometric analyses.

 f. **Reviewer’s query/comments**: There was no mention of how many replicates and trials were performed in physiological and biochemical experiments. I recommend mentioning the number of replicates and trials within replicates for the other experiments.

**Our answer:** We have added the number of biological replicates and technical replicates for all experiments under the Methods section.

 4. **Reviewer’s query/comments for the Result section:**

 a. **Reviewer’s query/comments**: Grey and white rows represent 50 µg and 100 µg of each antibiotic applied on the filter paper discs – please double-check Table 1.

**Our answer:** We have double-checked Table 1. The Table 1 legend is correct. Grey and white rows represent 50 µg and 100 µg of each antibiotic applied on the filter paper discs, respectively. Table 1 captions are correct in version 1 of the article. The numerical values in each row denote the diameters of zones of antibiotic-induced growth inhibitions. The diameter of the zone of growth inhibition is directly proportional to the sensitivity of the bacterium to the applied antibiotic. Hence, the larger the diameter of the zone of growth inhibition is, the more sensitive is the bacterium to the applied antibiotic and vice versa.  But we did find contradictory wordings and phrases in the text that describes Table 1 which might have confused the reviewer. We have revised the text description of Table 1. We hope this clarifies the concern of the reviewer.

 b. **Reviewer’s query/comments**: Authors stated: “We used polymyxin B amounts that were 1.4- fold to 2.8-fold higher than what is used for KB tests, and growth inhibition zones of Chlamydomonas and Clip185 were less than 13 mm (Table 1).” Why not use less than 12 mm instead of less than 13 mm to be consistent with a previous statement that used less than 12 mm as a reference?

**Our answer:** We have rectified the typographic error and have changed the growth inhibition zone diameter from 13 mm to 12 mm. Thanks for catching that mistake.

 c. **Reviewer’s query/comments**: In Figure 9, it could be helpful to add: Both Clip185 (B) and (D) were tested positive in the starch hydrolysis test as shown by a visible clear zone indicated by the arrows surrounding the growth (or if not an arrow perhaps encircle that area, so that the clear zone will be more easily seen by the readers).

**Our answer:** We have added black arrows in Figure 9B and Figure 9D to highlight the clear starch-hydrolyzed zone in these two figures. We have revised the Figure 9 legend and the respective result section accordingly.

 d. **Reviewer’s query/comments**: I recommend a revision to: This result strongly suggests the presence of decaprenoxanthin in Clip185. HPLC and/or LC/MS analyses could confirm the presence of decaprenoxanthin in Clip185. However, we do not have access to such analytical tools at our institution.

- Original statements were: This result very strongly indicates the presence of decaprenoxanthin in Clip185. We do not have access to such analytical tools at our institution.

**Our answer:**  We have accepted the reviewer’s suggestion and have revised the sentences under the Results section sub-section: Spectrophotometric analyses of pigments synthesized by Clip185.

 e. **Reviewer’s query/comments**: Please double-check the figures and the corresponding figure titles for Figures 15, 16, and 17. For example, Figure 15 has plates labelled Zinc but the title is Figure 15. Growth of Clip185 on LB media containing Cobalt.

**Our answer: ** We have uploaded correct Figures 15, 16, and 17 which match the respective figure legends in version 2. We did submit the correct figures/legends when we submitted version 1 of the article but during the publication process, for some reason the figures and legends got switched.

# Peer review: Reviewer response for version 1

**DOI:** 10.5256/f1000research.57195.r89080

**Published:** 2021-08-12

**Authors:** David Dewez

**Affiliations:** 1 Laboratory of Environmental and Analytical Biochemistry of Contaminants, Department of Chemistry, University of Quebec at Montreal, Montreal, QC, Canada

In this research work, the authors isolated and characterized a novel bacterial strain, named Clip185, which belongs to the genus *Microbacterium*. They showed that Clip185 is an aerobic, gram-positive rod, oxidase-negative, mesophilic, alpha-hemolytic bacterium. In particular, they demonstrated that Clip185 is antibiotic-resistant and heavy metal tolerant. Overall, this paper is well written and clearly explained. The methodology is also described in detail.

 The results are of high quality and well discussed. However, there are some mistakes that need to be corrected:

1. By reading the Table 1 caption, I understand that the grey rows represent 50 μg and the white rows 100 μg of each antibiotic applied, but I believe it is the opposite.
2. In Figure 6 caption, please add the abbreviation (TMPD) after ‘tetramethyl-p-phenylenediamine dihydrochloride’.
3. There is a mistake in the images showing the results of Figures 15, 16, and 17. They do not correspond to their respective captions. For example, Figure 15 shows the results of zinc which is supposed to be the ones of cobalt. It is very important to correct that.
4. In the Discussion section, at line 3: ‘As of 06-18-21’, what do you mean? I think you mean ‘As indicated in the NCBI genomic database last accessed on 6-18-2021’. Please correct.

Is the work clearly and accurately presented and does it cite the current literature?

Yes

If applicable, is the statistical analysis and its interpretation appropriate?

Yes

Are all the source data underlying the results available to ensure full reproducibility?

Yes

Is the study design appropriate and is the work technically sound?

Yes

Are the conclusions drawn adequately supported by the results?

Yes

Are sufficient details of methods and analysis provided to allow replication by others?

Yes

Reviewer Expertise:

Toxicology and chemistry of aquatic microorganisms.

I confirm that I have read this submission and believe that I have an appropriate level of expertise to confirm that it is of an acceptable scientific standard, however I have significant reservations, as outlined above.

# Response

**Published:** 2021-08-26

**Authors:** Mautusi Mitra

**Affiliations:** University of West Georgia, USA

Please see our point-by-point responses to your queries given below. Thanks for investing your time and energy to review our manuscript and providing us valuable inputs to improve the manuscript quality. Hope you will be willing to review our version 2 of the article that has addressed your comments.

**1. Reviewer's query/comment:** By reading the Table 1 caption, I understand that the grey rows represent 50 μg and the white rows represent 100 μg of each antibiotic applied, but I believe it is the opposite.

**Our answer:** Table 1 captions are correct in version 1 of the article. The grey rows represent 50 μg and the white rows 100 μg of each antibiotic applied. The numerical values in each row denote the diameters of zones of antibiotic-induced growth inhibitions. The diameter of the zone of growth inhibition is directly proportional to the sensitivity of the bacterium to the applied antibiotic. Hence, the larger the diameter of the zone of growth inhibition is, the more sensitive is the bacterium to the applied antibiotic and vice versa. But we did find contradictory wordings and phrases in the text that describes Table 1 which might have confused the reviewer. We have revised the text description of Table 1. We hope this clarifies the concern of the reviewer.

**2. Reviewer's query/comment: **In Figure 6 caption, please add the abbreviation (TMPD) after ‘tetramethyl-p-phenylenediamine dihydrochloride’.

**Our answer:** In Figure 6 caption, we have added the abbreviation (TMPD) after ‘tetramethyl-p-phenylenediamine dihydrochloride’ in version 2 of the article as per the suggestion of the reviewer.

**3. Reviewer's query/comment: **There is a mistake in the images showing the results of Figures 15, 16, and 17. They do not correspond to their respective captions. For example, Figure 15 shows the results of zinc which is supposed to be the ones of cobalt. It is very important to correct that.

**Our answer: ** We have uploaded correct Figures 15, 16, and 17 which match the respective figure legends in version 2. We did submit the correct figures/legends when we submitted version 1 of the article but during the publication process, for some reason the figures and legends got switched.

**4. Reviewer's query/comment:** In the Discussion section, at line 3: ‘As of 06-18-21’, what do you mean? I think you mean ‘As indicated in the NCBI genomic database last accessed on 6-18-2021’. Please correct.

**Our answer:** The List of Prokaryotic names with Standing in Nomenclature (LPSN) provides comprehensive information on the nomenclature of prokaryotes. As of 8-26-21, 124 species of *Microbacterium* have been identified with a validly published name in LPSN. We have clarified the sentence in line #3 under the Discussion section as per the reviewer’s suggestion by incorporating the above-stated information.
